# Parallel Visual Pathways with Topographic versus Nontopographic Organization Connect the *Drosophila* Eyes to the Central Brain

**DOI:** 10.1016/j.isci.2020.101590

**Published:** 2020-09-19

**Authors:** Lorin Timaeus, Laura Geid, Gizem Sancer, Mathias F. Wernet, Thomas Hummel

**Affiliations:** 1 Department of Neurobiology, University of Vienna, Vienna, Austria; 2 Center for Brain Research, Medical University of Vienna, Vienna, Austria; 3 Department of Biology, Freie Universität Berlin, Berlin, Germany

**Keywords:** Cellular Neuroscience, Molecular Neuroscience, Optical Imaging, Sensory Neuroscience

## Abstract

One hallmark of the visual system is a strict retinotopic organization from the periphery toward the central brain, where functional imaging in *Drosophila* revealed a spatially accurate representation of visual cues in the central complex. This raised the question how, on a circuit level, the topographic features are implemented, as the majority of visual neurons enter the central brain converge in optic glomeruli. We discovered a spatial segregation of topographic versus nontopographic projections of distinct classes of medullo-tubercular (MeTu) neurons into a specific visual glomerulus, the anterior optic tubercle (AOTU). These parallel channels synapse onto different tubercular-bulbar (TuBu) neurons, which in turn relay visual information onto specific central complex ring neurons in the bulb neuropil. Hence, our results provide the circuit basis for spatially accurate representation of visual information and highlight the AOTU's role as a prominent relay station for spatial information from the retina to the central brain.

## Highlights

• A *Drosophila* visual circuit conveys input from the periphery to the central brain
• Several synaptic pathways form parallel channels using the anterior optic tubercle
• Some pathways maintain topographic relationships across several synaptic steps
• Different target neurons in the central brain are identified

## Graphical Abstract

**Figure:**
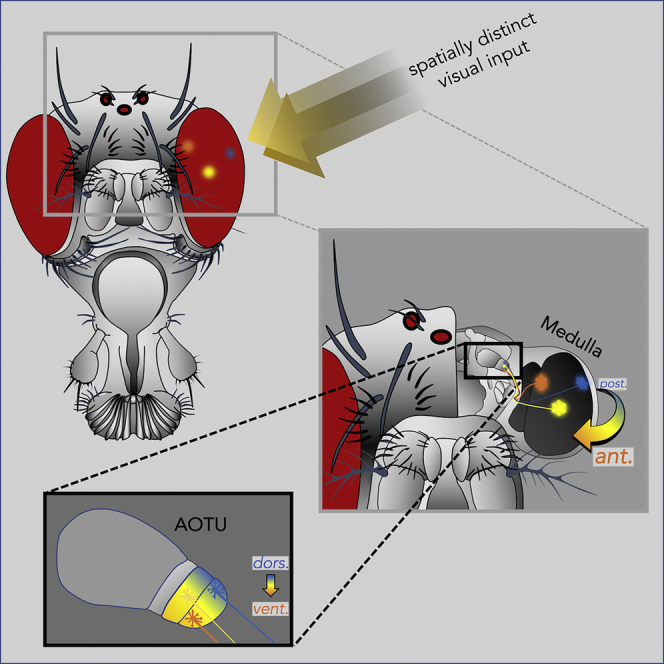

## Introduction

Most insects rely on visual cues for accurate maneuvering, which requires appropriate processing and fast integration of various visual stimuli (Egelhaaf and Kern, 2002; Heinze, 2017; Mauss et al., 2017). Fast decisions on whether to veer away from or approach an immobile or moving object while remaining able to quickly orientate within a complex, 3-dimensional environment are key tasks for their survival (Mauss et al., 2017). Research focused on dissecting neural circuits in the periphery of the visual system as well as in the central brain of a large variety of insect species, including the genetic model organism *Drosophila melanogaster*, has provided considerable insights into how information is processed beyond photoreceptor cells (Borst, 2014; Silies et al., 2014; Behnia and Desplan, 2015). Although the resolution of an insect compound eye does not rival that of a vertebrate retina (Kirschfeld, 1976), neuronal elements for the internal representation of certain features of the visual world have been successfully identified. Functional studies, more recently using genetically encoded effectors in *Drosophila*, have linked distinct structures of the visual system to processing discrete aspects of visual perception (Fisher et al., 2015; Schnell et al., 2010; Bahl et al., 2015; Ribeiro et al., 2018). Of special interest is the central complex (CX), a structure of interconnecting neuropils (named the protocerebral bridge, ellipsoid body, fan-shaped body, and noduli) located at the midline of the protocerebrum. Across insect orders, the CX's various functions comprise higher locomotor control, integration of multisensory input, representation of navigational cues, and different forms of memory formation (Strauss, 2002; Heinze and Homberg, 2007; El Jundi et al., 2014; Turner-Evans and Jayaraman, 2016; Varga et al., 2017; Liu et al., 2006; Ofstad et al., 2011).

The CX plays an important role in processing visual information in various insect orders, where neural pathways connecting the CX with the optic lobes have been characterized in hemi- and holometabolous insects (Homberg, 2015; Turner-Evans and Jayaraman, 2016; Honkanen et al., 2019; El Jundi et al., 2018; Franconville et al., 2018). In *Drosophila*, numerous studies using a variety of genetic tools described roles of the CX in visual pattern memory (Liu et al., 2006), encoding of visual experience and self-motion (Shiozaki and Kazama, 2017), flight-dependent visual responses (Weir and Dickinson, 2015), sun-guided navigation (Giraldo et al., 2018), and visual landmark recognition (Seelig and Jayaraman, 2015; Green et al., 2017), including sensorimotor remapping of visual information (Fisher et al., 2019), suggesting a substantial role of the CX in guiding object recognition for orientating in space. Although the neuroarchitecture of the *Drosophila* CX shows clear signs of a topographic organization (Lin et al., 2013; Franconville et al., 2018), the cellular composition and synaptic wiring diagram of neural circuits that relay spatial information from the optic lobes into the CX remain incompletely understood.

One prominent CX input pathway for visual information, with the ellipsoid body (EB) on the receiving end, has been identified as distinct classes of Ring neurons (R neurons), which form a stack of several ring-shaped layers in *Drosophila* (Hanesch et al., 1989; Wolff et al., 2015; Franconville et al., 2018). Afferent neurons are synaptically connected with R neurons via distinct microglomerular structures in the bulb neuropil adjacent to the EB (formerly referred to as the lateral triangle) (Ito et al., 2014). These connections are distributed retinotopically, because their positions correlate to small receptive fields on the ipsilateral side (Seelig and Jayaraman, 2013; Omoto et al., 2017). The transmission of spatial information from the optic lobes to the EB likely involves two synaptic neuropils: first, the R neuron dendrites in the bulb neuropil receive direct synaptic input from tubercular-bulbar neurons (or TuBu neurons), originating from the anterior optic tubercle (AOTU), one of several conserved optic glomeruli (Ito et al., 2014; Otsuna and Ito, 2006; Panser et al., 2016). Functional studies already described how R neurons inherit their receptive field properties from TuBu neurons (Sun et al., 2017; Shiozaki and Kazama, 2017). Secondly, distinct classes of medulla projection neurons (medullar-tubercular neurons or MeTu neurons) directly connect the medulla with the AOTU (Omoto et al., 2017; Otsuna et al., 2014). In contrast, the majority of remaining optic glomeruli are exclusively innervated by lobula columnar (LC) neurons (Otsuna and Ito, 2006; Wu et al., 2016). The AOTU is unusual among optic glomeruli in that it can be further subdivided into a medially located large unit (LU; also named AOTUm (Omoto et al., 2017), receiving input from the lobula via LC neurons) and a more lateral, small unit (SU, receiving input from the medulla via MeTu neurons). Although functional studies revealed that upon visual stimulation some optic glomeruli can be linked to specific behavioral responses, e.g. the detection of and response to small objects, escape, or reaching behavior (Keles and Frye, 2017; Wu et al., 2016), spatial information should be lost in the majority of optic glomeruli, due to convergence of intermingling LC inputs (Wu et al., 2016; Panser et al., 2016). However, other studies revealed that some LC afferents display some rough spatial restriction along the dorsoventral axis of the AOTU, indicating that a topographic pathway into the central brain may exist here (Wu et al., 2016). Hence, it remains unclear whether there is only a rough topographic representation of visual information along one spatial axis in the central brain or whether additional pathways with higher resolution also exist.

Here, we show that stereotyped topographic maps are built by distinct MeTu neuron subtypes within the SU of the AOTU, which is spatially separated from LC representation in the LU. Interestingly, the overlapping dendritic fields of different MeTu subtypes in the medulla diverge into multiple parallel visual channels that are subsequently maintained via parallel synaptic pathways from the AOTU to the bulb neuropil. Within the bulb, topographic channels connect with distinct receptive fields of CX ring neurons, whereas nontopographic channels have different R-neuron targets. Based on these data we propose a model in which specific domains of the AOTU form a central relay station for both topographic and nontopographic visual information, organized in multiple parallel channels, ideally suited for conveying distinct visual features to the central brain.

## Results

### Distinct Types of Afferent Arborizations within Optic Glomeruli

**Figure 1:**
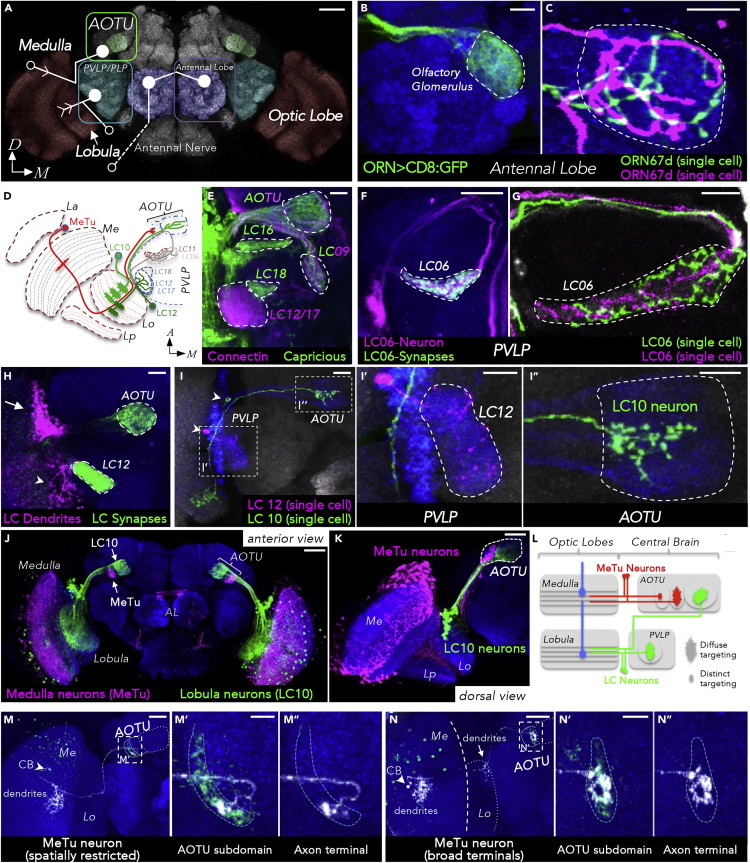
Organization of Afferent Projections within Olfactory and Optic Glomeruli (A) Overview over sensory glomeruli. Three pathways are shown, connecting medulla, lobula, and antenna with their respective target neuropils (for clarity, lobula-AOTU connections are not drawn). Open circles represent the position of the cell body, closed circles a target glomerulus, and arrows indicate dendritic arborizations. AOTU, anterior optic tubercle; PVLP, posterior ventrolateral protocerebrum; PLP, posterior lateral protocerebrum. Scale bar, 50 μm. (B and C) Axon terminals of OR67d-expressing olfactory receptor neurons in the antennal lobe are branching throughout their target glomerulus and intermingle with each other. Scale bars, 10 μm (C). (D) Schematic overview of visual projection neurons contributing to optic glomeruli (horizontal section). Only a subset of optic glomeruli is shown (the AOTU and five representatives in the PVLP). Afferents are illustrated by a single medullar (MeTu; red) and four lobular (LC; green, gray [terminals only]) neurons. Me, medulla; La, lamina; Lo, lobula; Lp, lobula plate. (E) Optic glomeruli are marked by combinatorial expression of different cell-adhesion molecules (Connectin, magenta; Capricious, green). Scale bar, 20 μm. (F) LC06 terminals (marked with syt:GFP) contribute to a characteristic optic glomerulus in the PVLP. Scale bar, 20 μm. (G) Two individual LC06 clones innervate the complete glomerulus. Scale bar, 10 μm. (H) Co-labeled LC10 and LC12 neurons. Somatodendritic (magenta) and presynaptic compartments (green) are labeled using DenMark and syt:GFP, respectively. Cell bodies of LC10 are marked with an arrow and LC12 with an arrowhead. Scale bar, 20 μm. (I) Single cell morphologies of LC10 and LC12. Although LC12 neurons branch throughout their target glomerulus (I′), LC10 neuron terminals are dorsoventrally restricted within the LU (I″). Arrowheads indicate position of cell bodies. (I′) and (I″) are magnified insets from (I). Scale bars, 20 μm (I); 10 μm (I' & I″). (J and K) AOTU compartments innervated either by MeTu or by LC10 neurons. Scale bars, 50 μm (J); 20 μm (K). (L) Schematic summary of pathways innervating AOTU and PVLP. Afferent medulla innervation indicated by blue neurons. (M and N) Single cell clones of MeTu cells with spatially restricted (M) or broad axon terminals (N). Different subtypes of MeTu neurons can be defined based on the position and size of terminal arborizations and whether the lobula is also innervated (arrow in (N)). The innervated area of the SU domains is magnified in the respective insets (M′, M″, N′, N″). CB, cell body. Scale bars, 20 μm (M and N); 5 μm (M′ and N′). For genotypes, see Supplemental Information.

Optic glomeruli and olfactory glomeruli are prominent neuropil structures located in different regions of the adult brain, with olfactory glomeruli concentrated within the antennal lobes of the deutocerebrum, whereas optic glomeruli form the AOTU, the posterior ventrolateral protocerebrum (PVLP), and the posterior lateral protocerebrum (PLP) (Figure 1A). To determine whether a common connectivity logic could be shared by olfactory and optic glomeruli, we investigated the arborization patterns of afferent fibers projecting into optic glomeruli. Olfactory glomeruli are characterized by a sensory class-specific convergence of afferent axons, each glomerulus thereby representing a unique odorant receptor identity (Laissue and Vosshall, 2008) (Figure 1B). Within each olfactory glomerulus, single sensory axon terminals arborize throughout the glomerular volume with all converging axon branches broadly overlapping and tightly intermingling (Hummel et al., 2003) (Figure 1C).

Inputs from LC neurons to optic glomeruli in the PLP/PVLP region are restricted to the ventrolateral brain region (Otsuna and Ito, 2006; Wu et al., 2016) (Figure 1D). In contrast, the more dorsally located AOTU receives afferent input via the anterior optic tract, containing both LC and MeTu fibers (Otsuna and Ito, 2006; Fischbach and Lyly-Hunerberg, 1983; Panser et al., 2016; Omoto et al., 2017) (Figure 1I). Using specific driver lines from the FlyLight and Vienna Tiles collection (Jenett et al., 2012; Kvon et al., 2014), a variety of LC neuron types could be identified and their class-specific segregation into single optic glomeruli visualized (Costa et al., 2016; Panser et al., 2016) (Figures 1F–1K). In analogy to work on olfactory glomeruli in the antennal lobe (Hong et al., 2009, 2012), we found that specific cell surface molecules are differentially expressed between different optic glomeruli (Figure 1E shows an example of the expression for Connectin and Capricious in different subsets of optic glomeruli).

To characterize afferent arborizations within optic glomeruli, we first generated single cell clones (see Transparent Methods for details) for different LC neuron types (LC06, LC10, LC12; Figures 1G and 1H). Similar to olfactory sensory neurons axon terminals, we found that each LC axon ramified throughout a single optic glomerulus and all neurons of the same LC class converged onto a common glomerular space (Figures 1G and 1H), thereby confirming the rather homogeneous arborization pattern within synaptic glomeruli in the PVP/PLVP neuropil (Wu et al., 2016). In contrast, a more diverse pattern of afferent innervation was observed in the AOTU large and small units (Figures 1D and 1J). Our systematic characterization of a large collection of AOTU-specific expression lines confirmed that the LU is the target field of LC neurons, whereas the SU is innervated by MeTu neurons (Figure 1J–L, and see below) (Panser et al., 2016; Omoto et al., 2017; Otsuna et al., 2014). Single LC afferent terminals in the LU arborized throughout large areas of the glomerular subunit's volume, with some enrichment in the dorsal versus ventral regions of the LU (Figure 1I’’) (Wu et al., 2016). In contrast, single MeTu afferents in the SU were more variable, ranging from broad (in close proximity to the LU) to spatially restricted in more lateral regions (Figures 1M, 1N, S1, S3, and S4), indicating that different MeTu classes for distinct spatial representation might exist within the AOTU. This structural feature of spatially restricted afferent terminals makes the SU of the AOTU a candidate for a neuropil that could maintain topographic representation of visual information within the central brain.

### Morphological and Molecular Domain Organization of the AOTU

**Figure 2:**
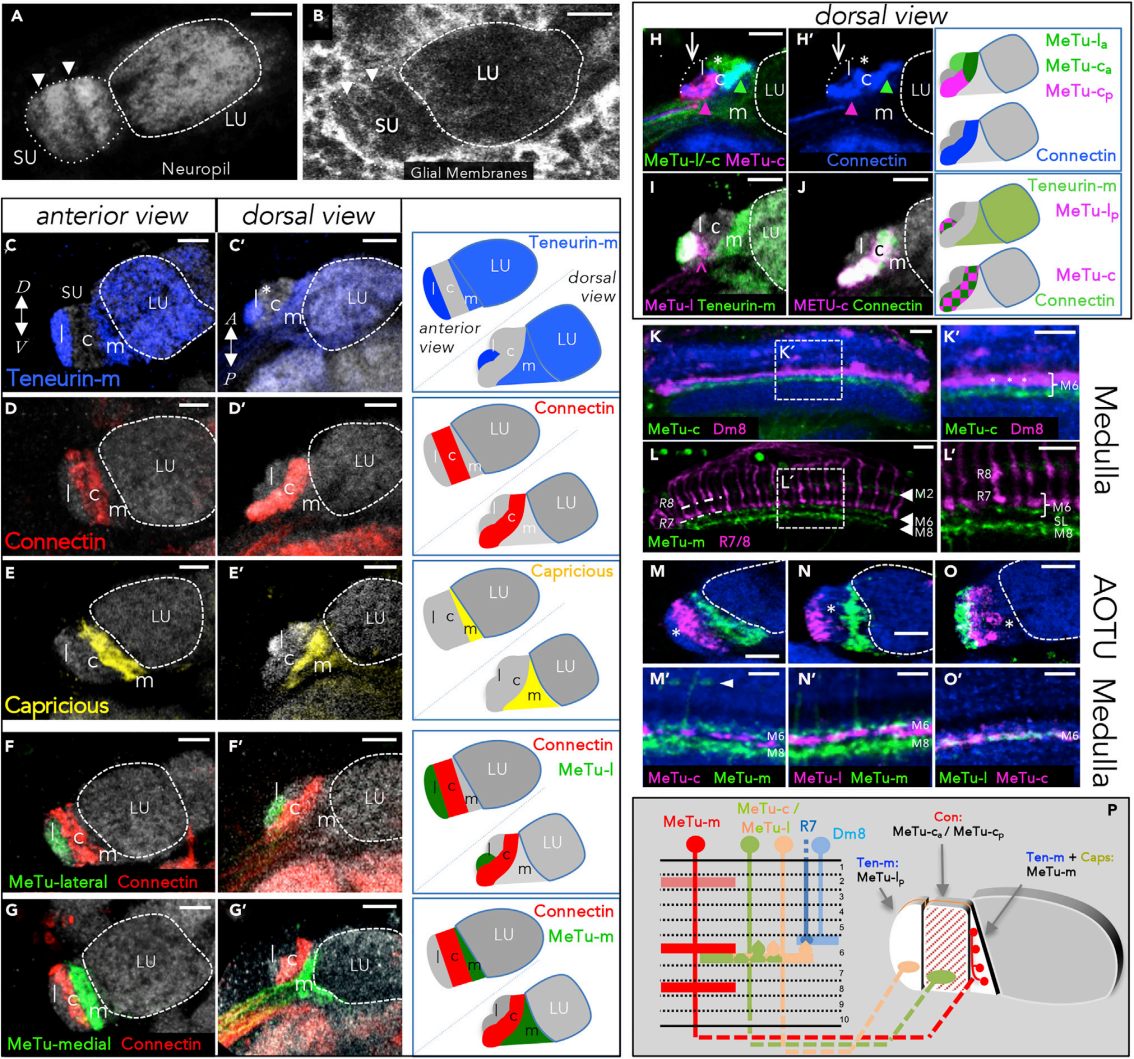
Classification of MeTu Neuron Subtypes All scale bars, 10 μm. (A) Subdivision of AOTU's small unit (SU) can readily be observed with neuropil markers (anti-CadN). Arrowheads indicate borders of subdomains. In contrast, the large unit (LU) has a uniform appearance. (B) Glial labeling using repo-Gal4 reflects the compartmentalization of the AOTU's SU (arrowheads). (C–E) Each SU domain is characterized by a unique combination of three cell-adhesion molecules: Teneurin-m (blue) is strongly expressed in the lateral domain (C), with lower intensity in the medial domain and the LU. The lateral domain is further divided into an anterior, Teneurin-m-negative (asterisk) and a posterior, Teneurin-m-positive compartment (C′). Connectin expression (red) defines the central domain (D and D′). Capricious-Gal4 (yellow) marks the medial domain (E and E′). Different brains were taken for the respective anterior and dorsal views. (F and G) Domain borders are respected by terminals of MeTu subtypes: different Gal4-labeled MeTu neurons innervate either the lateral (F–F′) or medial domain (G–G′), without overlapping into the central, Connectin-positive (red) domain. Different brains were taken for the respective anterior and dorsal views. (H–J) Further division of the lateral and central domain into anterior and posterior compartments: a combination of LexA- (green) and Gal4- (magenta) lines reveals a subdivision of the central domain (H). A small subset of LexA-expressing neurons also innervates the anterior part of the lateral domain (asterisk). Same brain as in (H) without the endogenous signal; anti-Connectin (blue) labels the complete central domain (H′). The posterior part of the lateral domain is exclusively innervated by a population of MeTu-l neurons and likewise defined by Teneurin-m expression (green) (I). The arrowhead marks turning MeTu-l axons (these are not innervating the central domain). The complete central, Connectin-positive (green) domain is labeled by a line specific for MeTu-c neurons (magenta) (J). (K and K’) Dendrites of MeTu-c neurons (green) are restricted in medulla layer M6, in a sublayer below R7 terminals and Dm8 neurons (magenta). Magnified inset in (K′). (L and L’) Three medulla layers are occupied by MeTu-m (arrowheads). Magnified inset in (L′). Photoreceptors are labeled with anti-Chaoptin (24B10). SL, serpentine layer. (M–O’) MeTu-c/-l neurons and MeTu-m neurons do not overlap in the medulla (M′–O′). Asterisks indicate the respective unlabeled SU-domain. MeTu-c and MeTu-l terminals are separated in the SU, while sharing the same medulla layer. For each column, the same brain has been used to show both AOTU and medulla pattern, respectively. Arrowhead in (M′) points to MeTu-m dendrites in M2. (P) Schematic overview over MeTu neuron subtype morphology in medulla and SU. Caps: Capricious; Con: Connectin; Ten-m: Teneurin-m. For genotypes, see Supplemental Information.

To determine how the architecture of the AOTU correlated with patterns of afferent innervation, we first co-labeled glial membranes with the neuropil epitope N-cadherin (Figures 2A and 2B). As previously reported (Omoto et al., 2017), a subdivision of the SU neuropil into multiple domains along the medial-lateral axis became visible, whereas the LU appears like a homogeneous neuropil without any obvious morphological substructures (Figures 2A and 2B). This organization of the SU neuropil into several subdomains was further supported by the combinatorial expression pattern of various cell adhesion molecules. For example, we found the synaptic cell adhesion molecule Teneurin-m to be broadly expressed throughout the AOTU neuropil with the exception of the central subdomain of the SU (SU-c) and the anterior part of the lateral SU (SU-l) (Figure 2C). On the other hand, the adhesion molecules Connectin and Capricious were specifically expressed in the SU-c and medial SU (SU-m) domains, respectively (Figures 2D–2G). We then tested whether the SU subdomains matched different classes of MeTu afferents (Figures 2H–2J). Based on the terminal arborization patterns from 13 independent expression lines (see Transparent Methods) we could distinguish at least 3 distinct, nonoverlapping populations of MeTu neurons. Based on the segregation of their axons within the AOTU, these neurons were classified as MeTu-lateral (-l), MeTu-central (-c), and MeTu-medial (-m) (compare Figures 2M–2O) (see discussion for a related description by (Omoto et al., 2017)). A more detailed analysis of molecular markers in combination with MeTu expression lines revealed a further subdivision of the lateral SU domain (SU-l) into distinct anterior and posterior subdomains (SU-l_a_ versus SU-l_p_, Figures 2C′ and 2F′), which was not apparent for the LU (Figures 2C′, 2D′, 2E′, and 2G′). Furthermore, by combining independent Gal4 and LexA expression lines, a similar anteroposterior division of the central SU domain (SU-c) into SU-c_a_ and SU-c_p_ subdomains was found (Figure 2H). Importantly, the terminals of specific MeTu driver lines co-labeled specifically with neuropil markers defining these specific subdomains of the SU, indicating that specific subdomains are indeed targeted by specific MeTu classes (Figures 2I and 2H′). In contrast, other expression lines labeled a broader set of neurons innervating more than one subdomain (Figure 2J).

To get further insights into the neuronal identity of the different MeTu populations, we visualized their dendritic arborizations in the medulla neuropil (Figures 2K–2O′). Interestingly, all three MeTu classes formed dendrites in medulla layer M6, where the UV-sensitive R7 photoreceptor cells target their main synaptic partner, the distal medulla cell type Dm8 (Karuppudurai et al., 2014; Ting et al., 2014; Gao et al., 2008; Nern et al., 2015). However, MeTu dendrites were located below the terminals of R7 cells and therefore separated from the R7/Dm8 synaptic area (Figures 2K and 2L). For the majority of MeTu-l and MeTu-c neurons, the M6 layer appeared to be the only layer with dendritic signal (Figures 2K and 2O′). In contrast, MeTu-m neurons formed dendritic arborizations in two additional medulla layers located both proximal and distal to layer M6, most likely layer M2 and layer M8 (Figures 2L, 2L′, 2M′, and 2N′). Interestingly, in M6, MeTu-m dendrites segregated from MeTu-l/-c dendrites (Figures 2M′ and 2N′), thereby revealing three distinct sub-layers within this medulla layer (R7/Dm8, MeTu-m, MeTu-l/-c) (Figure 2P). In summary, the AOTU receives direct input from distinct types of MeTu neurons, which differ in their dendritic layering, target subdomain, and molecular identity (summarized in Figure 2P).

### Photoreceptor Connectivity of MeTu Subtypes

**Figure 3:**
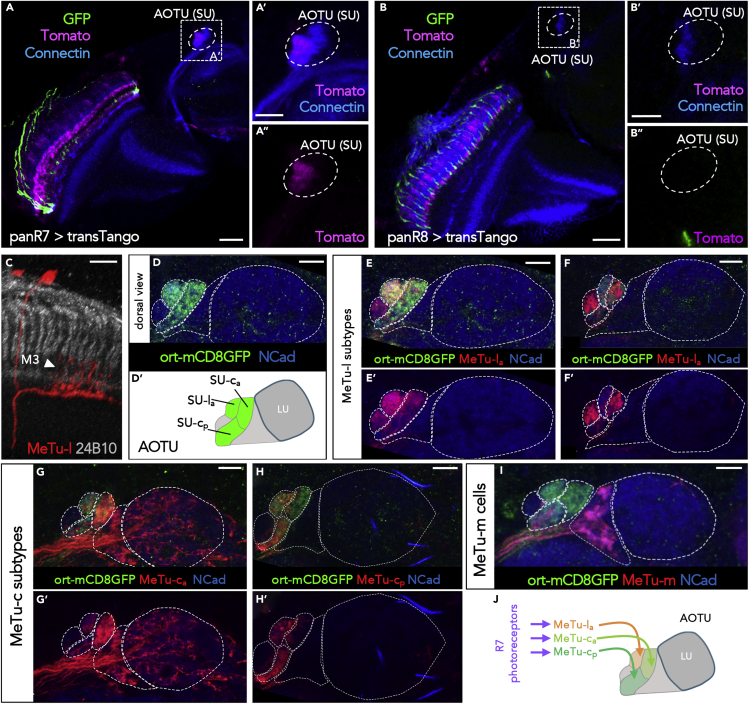
Connectivity between Photoreceptors and MeTu Neurons (A) R7-specific transTango experiment using (rh3+rh4)-Gal4 (‘panR7’) reveals tomato-positive transTango signal in MeTu processes to the SU of the AOTU (dashed area in the magnified inset; A′ and A″). Scale bars, 20 μm (A); 10 μm (A′). (B) No transTango signal is detectable in (rh5+rh6/‘panR8’) > transTango experiments (B′ and B″). Scale bars, 20 μm (B); 10 μm (B′). (C) Single cell MeTu-l clone visualized via R94G05 > MCFO-1 reveals an exemplary neuron with dendrites in multiple medulla layers and processes reaching to higher medulla levels (arrowhead in layer M3). Scale bar, 10 μm. (D) Expression of the newly generated ort-mCD8:GFP transgene in the AOTU. The domains of the SU are labeled (SU-l_a_, SU-c_a_, SU-c_p_), whereas the LU is not labeled (D′). Scale bar, 10 μm. (E) MeTu-l driver R94G05 labels both MeTu-l_a_ and MeTu-l_p_ populations, yet only MeTu-l_a_ are postsynaptic to photoreceptors (E’; without GFP-signal). Scale bar, 10 μm. (F) MeTu-l driver R52H03 specifically labels MeTu-l_p_ and MeTu-c_a_ populations, of which only MeTu-c_a_ are postsynaptic to photoreceptors (F’; without GFP-signal). Scale bar, 10 μm. (G) MeTu-c driver R67C09 specifically labels MeTu-c_a_ cells, which are postsynaptic to photoreceptors (G’; without GFP-signal). Scale bar, 10 μm. (H) MeTu-c driver R25H10 specifically labels MeTu-l_a_ and MeTu-c_p_ populations, both of which are postsynaptic to photoreceptors (H’; without GFP-signal). Scale bar, 10 μm. (I) MeTu-l driver R20B05 labels MeTu-m cells, which are not postsynaptic to photoreceptors (A′). Scale bar, 10 μm. (J) Schematic summary of the results from (D–I). For genotypes, see Supplemental Information.

To investigate whether direct synaptic contacts between MeTu-l/-c dendrites in layer M6 and inner photoreceptors R7 (and less likely R8) might exist, the transsynaptic tracer “transTango” (Talay et al., 2017) was expressed under the control of either R7- or R8-specific rhodopsin-Gal4 driver combinations, respectively (Figures 3A and 3B; see Transparent Methods for details). Significant labeling of the SU was detected following the transTango expression in R7 (A′), whereas no signal was detected in the AOTU in the case of R8 > transTango (B′). In the former case, the obtained patchy signal indicated that only UV-sensitive R7 cells are indeed synaptically connected to some, but probably not all, MeTu-l/-c neurons. Although dendrites of MeTu-l and MeTu-c cells were mostly restricted to medulla layer M6, we noticed that some MeTu cell clones formed vertical processes reaching beyond medulla layer M6 (almost reaching M3), thereby making R7 photoreceptor → MeTu synapses a possibility (see MeTu-l clone in Figure 3C). In order to systematically test which MeTu subtypes could be postsynaptic to R7 photoreceptors, we generated a transcriptional fusion of a ∼3.5 kb fragment containing the promoter sequences of the histamine receptor Ort, driving expression of membrane tagged mCD8:GFP (see Transparent Methods for details). Because histamine is the neurotransmitter expressed by all insect photoreceptors (Stuart, 1999), many of their synaptic targets should be marked by Ort expression (Gao et al., 2008). As expected, this ort-mCD8:GFP transgene labeled many cell types throughout the optic lobes as putative photoreceptor targets (Figure S2), including MeTu axon projections into discrete domains of the AOTU (Figure 3D). Out of the five domains of the SU, only three were clearly positive for ort-mCD8:GFP, namely SU-l_a_, SU-c_a_, and SU-c_p_. We therefore proceeded to confirm that processes from MeTu subtypes terminating in these domains indeed co-labeled with GFP, using a combination of different subdomain-specific drivers. Out of both MeTu-l subtypes, only axons of MeTu-l_a_ neurons co-labeled with GFP, whereas MeTu-l_p_ did not (Figures 3E and 3F). In contrast, axons from both MeTu-c subtypes (c_a_ and c_p_; both individually labeled using different driver lines) co-labeled with GFP (Figures 3G–3H). Finally, axons of MeTu-m cells never co-labeled with GFP (Figure 3I). In summary, of all MeTu cells innervating the SU of the AOTU, only MeTu-l_a_, MeTu-c_a_, and MeTu-c_p_ were identified as potential synaptic targets of R7 photoreceptors (Figure 3J).

### Topographic Organization of AOTU Afferents

**Figure 4:**
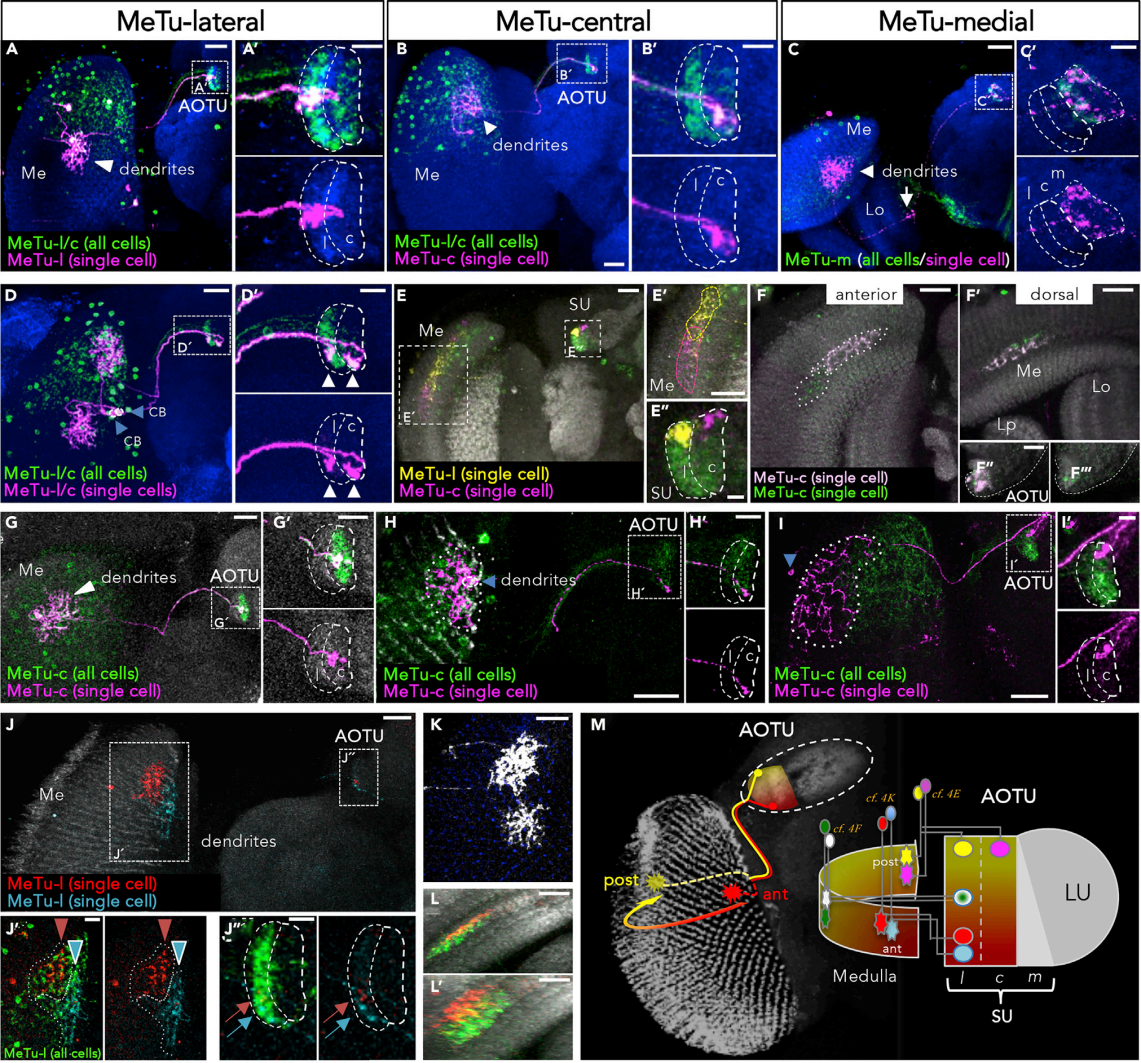
Topographic Organization of AOTU Projections (A–C) FLYBOW-labeling of MeTu-neurons innervating their respective domain of the SU (magnified in A′–C’; without GFP-signal in the lower row). Arrow in (C) indicates innervation of the lobula by MeTu-m neurons. Scale bars, 20 μm, (A–C); 10 μm (A′–C′). (D) Two neighboring cells (blue arrowheads) innervate different positions within the dorsal medulla and target the lateral and the central SU-domain, respectively (white arrowheads). Magnified inset of the SU domain in (D′). CB, cell body. Scale bars, 20 μm (D); 10 μm (D′). (E) Two MeTu clones with overlapping dendritic fields at the posterior edge of the medulla target to the dorsal edge of either the lateral domain (yellow neuron) or the central domain (magenta neuron), respectively. Magnified inset of the medulla in (E′) and of the SU in (E″). Scale bars, 20 μm (E and E′); 5 μm (E″). (F) Anteroposterior, but not dorsoventral, positions in the medulla correlate with topographic projections in the AOTU: MeTu-c neurons at the same a-p position in the medulla target into the same area of the central SU-domain (the brain has been rotated between (F) and (F′)). AOTU of the same brain shown in (F″, F‴), with only one cell labeled in (F‴). Scale bars, 20 μm (F and F′); 5 μm (F″). (G–I) Topographic projections of MeTu-c neurons: central dendritic fields in the medulla correlate with central termination the AOTU (G′), anterior dendritic positions in the medulla correlate with ventral targeting (H′), whereas posterior medullar dendrites correlate with dorsal termination (I′). Size of dendritic fields and size of innervated target area did not correlate (blue arrowheads indicate cell bodies). Scale bars, 20 μm (G, H, and I); 5 μm (magnified insets G′, H′, and I′). (J) Dendritic fields of neighboring clones at the anterior rim of the medulla maintain their topography in the AOTU: the red clone, being located more posteriorly in the medulla, terminates at a more dorsal position in the AOTU. Magnified inset of the medulla in J′ and the SU in J’’. Scale bars, 20 μm (J); 10 μm (J′ and J″). (K) The size of dendritic fields varies among MeTu-l neurons. Scale bar, 20 μm. (L) Overlap of dendritic fields between two MeTu-l clones (brain has been rotated between L and L′). Scale bars, 20 μm. (M) Summary of the FLYBOW-pairs described above (colors accordingly) and model of topographic relationships between medulla dendritic fields and SU axis of innervation. For genotypes, see Supplemental Information.

Next, we proceeded to a more systematic characterization of how AOTU subdomains correlate with MeTu neuron identity at a single cell level. Clonal analysis revealed a stereotypical, subtype-specific pattern of MeTu innervation, where any given MeTu axon terminates in only one of the five SU subdomains (Figures 4A–4C). For MeTu-l and MeTu-c neurons, a spatially restricted termination pattern was observed in their respective SU subdomains (Figures 4A and 4B). In contrast, afferent arborizations of MeTu-m cells extended throughout a large portion of their compartment (Figures 4C and S1), resembling the previously published projection pattern of LC10 neurons in the LU (see Figure 1I). The differences between MeTu-m neurons (with dendritic arborizations in multiple medulla layers and axonal convergence throughout their SU subdomain) versus MeTu-l + MeTu-c neurons (with dendrites restricted to medulla layer M6 and spatially restricted axon terminals in the AOTU) therefore support the existence of morphologically and functionally distinct visual channels into the central brain.

Dendritic fields of single MeTu-neurons always covered multiple medulla columns, yet the specific field size of individual MeTu-neuron clones varied considerably: at the anterior and posterior medulla border, neurons can be found that stretch across a major part of the dorsal medulla, either covering a large dendritic area in both axes (Figure 4I) or spreading along the medulla border with limited a-p dimension (compare first two images in Figure S5). In the central part of the medulla, dendrites of MeTu neurons are more circularly shaped, ranging from ∼20 medulla columns covered (lower cell in Figure 4K) to >50 columns (Figure S4; marked with an asterisk). Importantly, the differential labeling of randomly induced two-cell clones for either MeTu-l or MeTu-c neurons (using FLYBOW (Hadjieconomou et al., 2011), see Transparent Methods) manifested two crucial features with regard to the spatial organization of their terminals in the AOTU: first, MeTu neurons of the same type (l/l or c/c) with neighboring dendritic fields in the medulla always projected to adjacent positions in the corresponding SU domain (Figure 4J). Secondly, MeTu neurons of different types (l/c) with overlapping dendritic fields in the medulla always projected to the same position along the d-v axis, yet in adjacent SU domains (Figure 4E). To determine whether MeTu-l and MeTu-c cells innervated their corresponding SU domain in a topographic fashion, we correlated their relative position of dendrites in the medulla with their axon terminals and AOTU, respectively (Figures 4G–4I). For both cell types we could observe a strict correlation between the dendritic position along the anteroposterior (a-p) axis in the medulla and the axonal termination point along dorsoventral (d-v) axis in the AOTU (Figure 4M, n = 35) (Figures S3–S5). According to this wiring scheme, MeTu-l and MeTu-c neurons with dendrites at the anterior rim of the medulla neuropil target the most ventral position in their corresponding SU domain, whereas neurons with dendrites at the posterior rim of the medulla connect to a dorsal edge of the SU (Figures 4H and 4I). Furthermore, MeTu-(l/c) clones with dendrites in more medial medulla regions also targeted into medial position in the AOTU (Figure 4J). The spatial arrangement of MeTu dendrites along the d-v axis of the medulla was not converted into a topographic targeting pattern along the a-p axis in their SU domains (Figures 4F–4F‴). The broader innervation pattern of many MeTu-m terminals in their respective domain is very different from the other MeTu classes, yet we cannot exclude that some MeTu-m neurons with more restricted terminals also form a topological arrangement (Figure S1). In summary, these data revealed the structural organization of a topographic representation in the AOTU in which different MeTu cell types form multiple parallel channels from the medulla to a central brain.

### AOTU Efferents Maintain Domain Identity and Visual Topography

**Figure 5:**
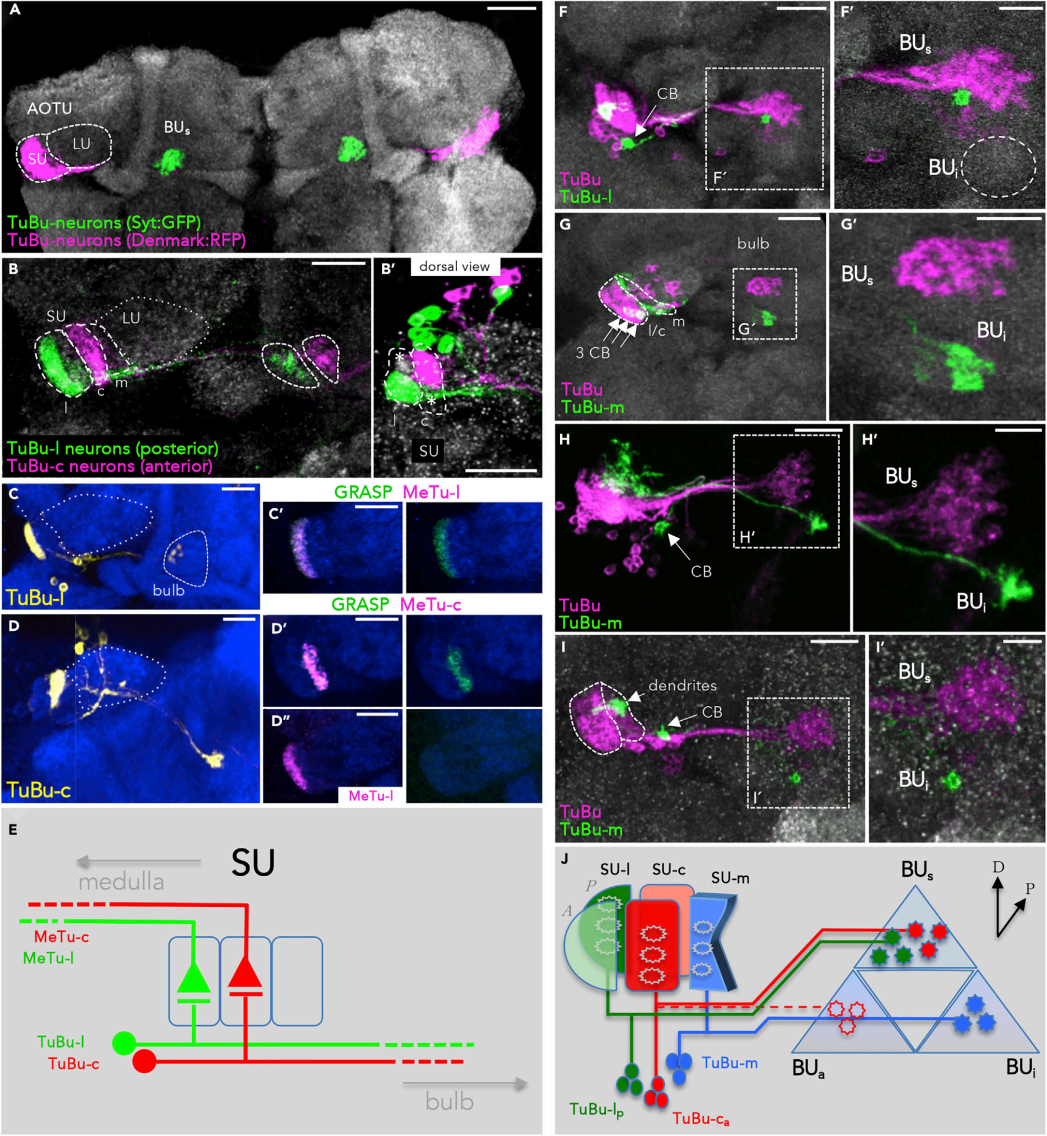
Bulb-Innervating Neurons Descending from the AOTU Maintain Domain Identity (A) The bulb neuropil receives input from all three SU-domains. Scale bar, 50 μm. (B) Terminals of TuBu-l and TuBu-c neurons are spatially separated within the bulb. Dorsal view of a different brain in B’; asterisks mark the unlabeled anterolateral and posterior-central SU-domains. Scale bars, 20 μm. (C and D) Pre- to postsynaptic matching of domain-specific expression lines in the SU revealed by synGRASP. Anti-GFP (yellow) detects the presynaptic moiety of TuBu-l, expressed under Gal4-control (C). Positive GRASP-signal is obtained in combination with MeTu-l neurons (C′). TuBu-c neurons (yellow) are synaptic partners of MeTu-c neurons (D′), whereas no synaptic connections are formed with MeTu-l neurons (D″). Scale bars, 20 μm. (E) Scheme depicting how afferent MeTu neurons and efferent TuBu neuron subtypes form circuits in their respective SU-domains. (F–I’) FLYBOW-labeling using a reporter for the majority of TuBu neurons. TuBu innervations are virtually absent from the BU_i_ (dashed circle). CB, cell body. TuBu-l dendrites and axonal terminals are spatially restricted (F′). Three TuBu-m clones innervate a ventral area in the bulb (BU_i_), separate from TuBu-l and -c neurons (G′). TuBu-m arborization size is variable both in AOTU and bulb, ranging from covering larger areas (H) to spatially restricted (I). Scale bars, 20 μm (F, G, H, and I); 10 μm (magnified insets F′, G′, H′, I′). (J) Schematic describing the distribution of three TuBu classes in the bulb neuropil. The innervation of the BU_a_ has not been analyzed in this study. For genotypes, see Supplemental Information.

If the AOTU served as a relay station of spatial information from the optic lobes to central integration centers of the brain, one would expect a matching pattern of connections between MeTu subtypes and corresponding AOTU output neurons along the d-v axis, at least for the lateral and central SU domains. We and other groups identified a large set of expression lines for AOTU projection neurons targeting the bulb region (TuBu neurons) (Omoto et al., 2017; Sun et al., 2017; Shiozaki and Kazama, 2017). An overview of the pathway is given in Figure 5A. These TuBu expression lines show domain-specific restriction of their dendritic fields, corresponding to the SU-l, -c, and -m domains and were therefore classified as TuBu-l, -c, and -m neurons, respectively (Figures 5B and 5E; compare also (Omoto et al., 2017)). In accordance with segregation of MeTu neurons in a-p axis in the SU (Figures 2H and 2I), this pattern could also be observed for the corresponding TuBu neurons (Figure 5B’). The dendritic field size of TuBu single cell clones matched the extent of axonal arborizations from corresponding MeTu cells. In agreement with subdomain-specific connectivity, TuBu-l and -c domains manifested the most restricted dendritic arbors, whereas TuBu-m neurons formed broad dendritic fields (Figures 5H and 5I). We counted an average number of 8–12 TuBu neurons for different classes, covering a given SU domain along the d-v axis. To test if the spatial overlap of MeTu axon terminals and TuBu dendrites was indicative of synaptic connections we used the activity-dependent GRASP technique (Karuppudurai et al., 2014; Macpherson et al., 2015). Indeed, GRASP between presynaptic MeTu neuron subtypes and various sets of TuBu neurons revealed a strict matching of synaptic partners within but not across SU domains (Figures 5C–5D″).

### Nonstereotypic Organization of AOTU Efferents in the Bulb Region

**Figure 6:**
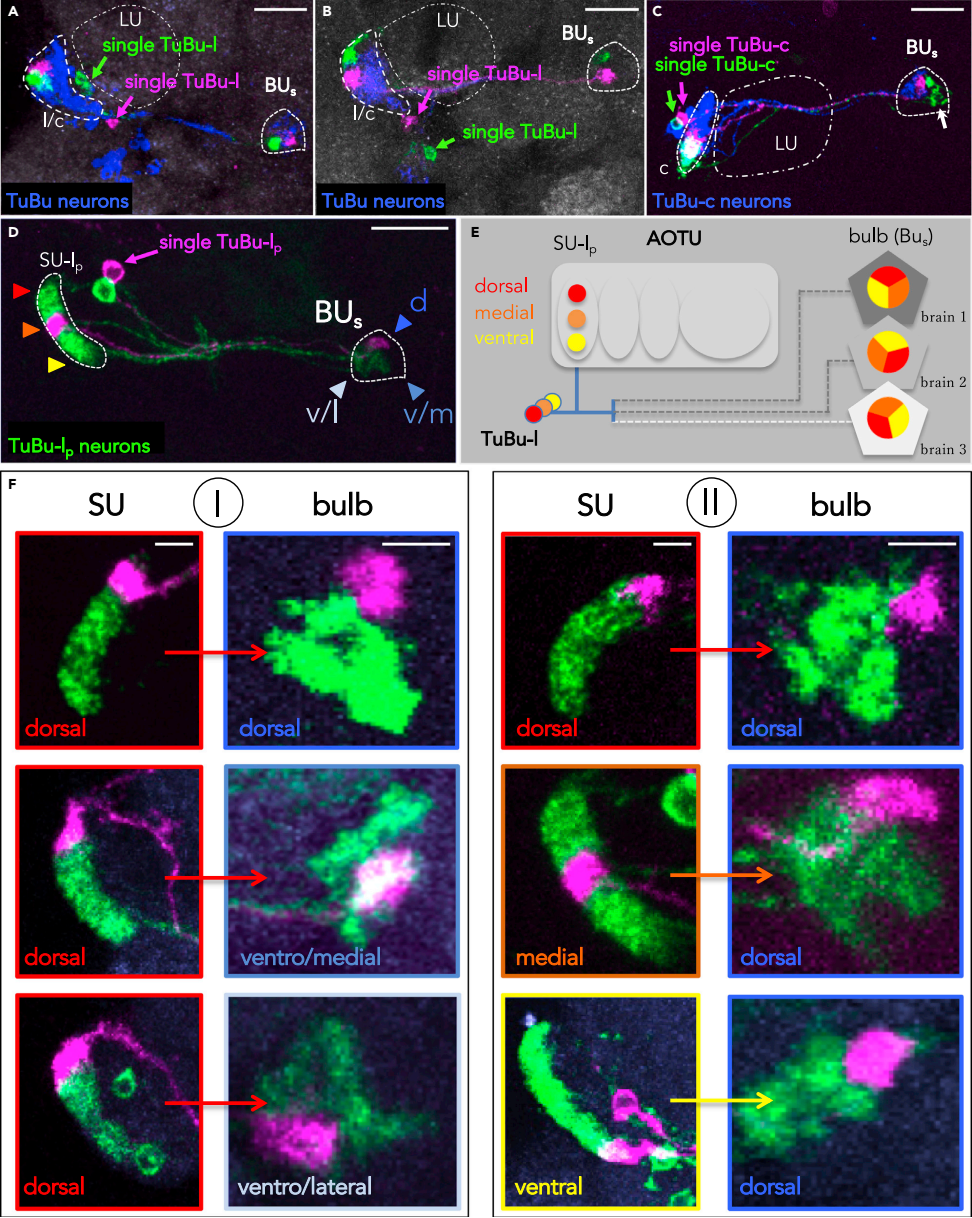
Variability of Innervation Patterns across TuBu Neurons (A–C) The axon terminals of neighboring TuBu-l neurons maintain their proximity in the bulb, but their orientation is variable, both when labeling all TuBu neurons (A,B) and TuBu-c specifically (C). Scale bars, 20 μm. (D) Example of an FLYBOW-induced TuBu-l single cell clone (magenta) while co-labeling all TuBu-l_p_ neurons (green). Color coding of arrowheads indicates dorsoventral distribution in the AOTU as well as positions in the BU_s_ (dorsal, ventrolateral, ventromedial), same as in subsequent panels. Scale bar, 20 μm. (E) Schematic depicting the lack of stereotypic orientation of terminals from adjacent TuBu-l_p_ neurons in the bulb. (F) There is no topographic correlation between dendritic position in the AOTU and target field in the bulb. Neurons with dorsal positions in the AOTU target to various positions within the lateral sector of the BUs (column I). Likewise, a similar position in the bulb are innervated from various positions along the d-v axis in the AOTU (column II). For genotypes, see Supplemental Information. Scale bars, 5 μm.

TuBu axons form a single fascicle that extends from the AOTU toward the bulb, where they subsequently segregate toward distinct domains according to their SU domain identity (Figure 5J; compare also Omoto et al., 2017). We found that TuBu-l and -c neurons terminated in adjacent regions of the superior bulb (BU_s_), whereas axons of TuBu-m neurons targeted into the inferior bulb (BU_i_) (Figures 5F′, 5G′, 5H′, and 5I′). Hence, topographic and nontopographic visual pathways remain spatially segregated within the bulb (we did not analyze innervations of the SU_a_, described in Omoto et al., 2017). We next analyzed the spatial organization of dendritic and axonal arborization of single cell and small size TuBu clones. To determine if the retinotopic representation from the AOTU is translated into the terminals of TuBu cells within the bulb region, we compared the relative positions of TuBu dendrites in the SU with the location of their axon terminals in the bulb region by generating two-cell clones within a population of TuBu-l and TuBu-c neurons, respectively (Figures 6A–6C). This analysis revealed that adjacent dendritic positions in the AOTU are indeed maintained within neighboring domains of the bulb, although their relative position to each other within the bulb area is variable (Figures 6A and 6B). To further characterize the spatial patterning of TuBu neurons, we generated a series of single cell clones and compared the relative position of TuBu dendrites in the SU with their axon termination areas in the bulb, this time for individual TuBu clones (Figure 6D). In contrast to the strict spatial correlation between MeTu neuron dendrite position along the a-p axis and its axon termination along the d-v axis, the position of TuBu dendritic fields within the SU domain did not predict their site of axon termination within the bulb area (Figures 6E and 6F). For example, single TuBu-l clones with dendritic fields in the dorsal SU domain manifested projections either to the dorsal, ventrolateral, or ventromedial bulb domains (Figure 6F, left column). Similarly, the dorsal bulb region could receive TuBu afferents from neurons with either dorsal, medial, or ventral SU positions (Figure 6F, right column). Given the fixed spatial proximity of TuBu axon terminals with adjacent dendritic fields described earlier, these data suggest that the topographic map of the AOTU is maintained in the bulb where it translates into a more variable organization regarding the a-p and d-v axes of TuBu terminals within a sector of the bulb.

### Projections of AOTU Domain Identity onto Ring Neurons of the EB

**Figure 7:**
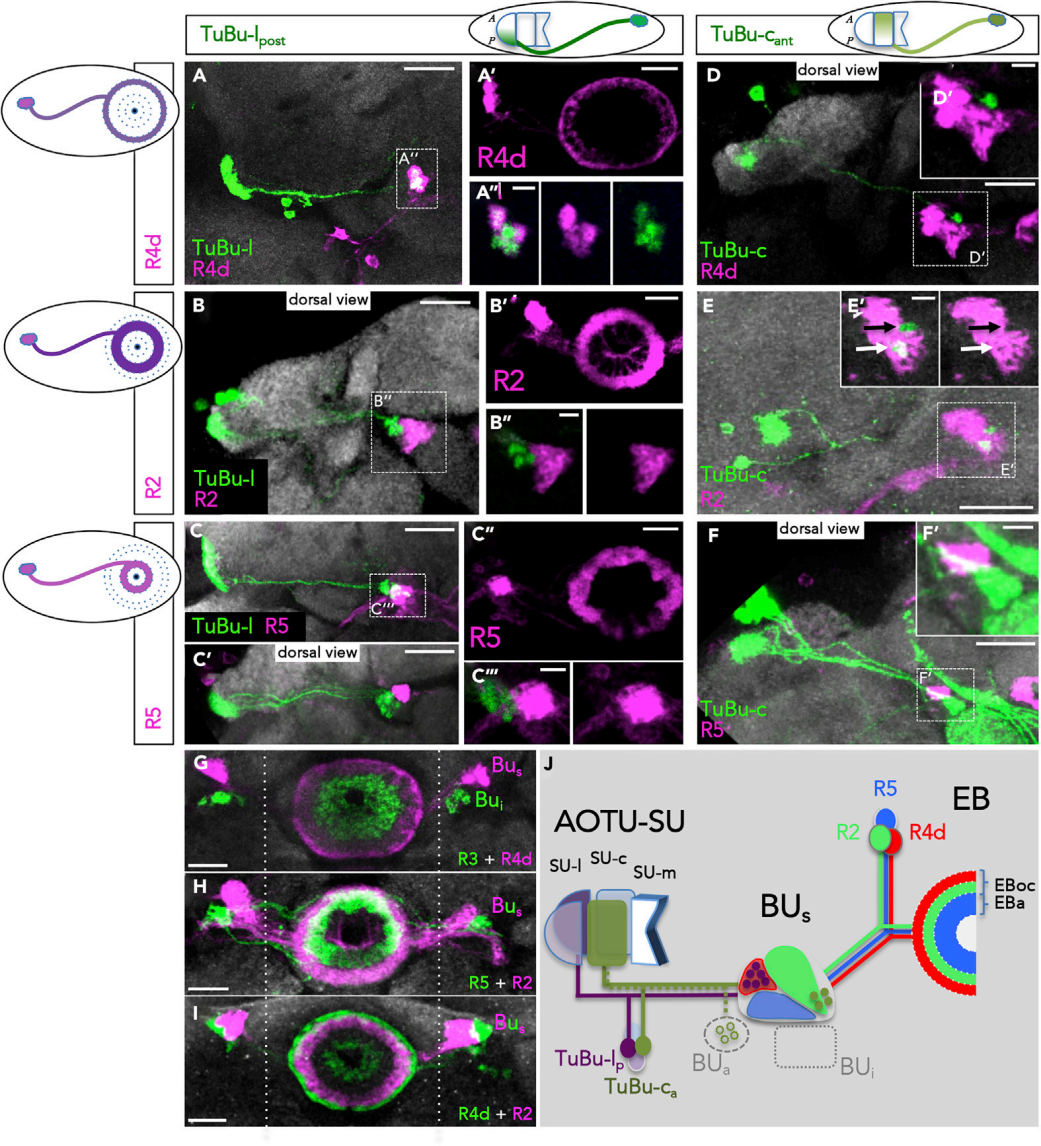
Distinct AOTU Pathways Connect with Specific R Neuron Classes In the BU_s_, different TuBu classes connect to a set of R neurons. Two LexA expression lines label the posterior lateral domain and the anterior central domain of the SU, respectively. The BU_a_ and BU_i_ are not covered in this analysis. (A–C) TuBu-l_p_ neurons innervate the BU_s_, where they exclusively contact R4d neurons (A and A″), but not R2 (B) or R5 (C) neurons. Scale bars, 20 μm (A, A′, B, B′, C-C″); 5 μm (A″, B″, C‴; magnified insets with reduced stack size). (D–F) TuBu-c_a_ neurons partially overlap with R2 neurons (E) but not with R4d (D) or R5 (F). White and black arrows in (E) indicate the presence or absence of co-labeling of expression lines, respectively. Scale bars, 20 μm (D–F); 5 μm (magnified insets D′–F′). (G–I) Co-labeling of R neurons reveals the coverage of different fields within the BU. R3 neurons do not contribute to the BU_i_. Scale bars, 20 μm. (J) Proposed segregation of visual information of TuBu-l_p_ and TuBu-c_a_ neurons in the superior bulb. Innervation of the BU_a_ in reference to Omoto et al. (2017). Filled dark stars in the BU_s_ indicate terminal endings of TuBu neurons (microglomeruli). EBoc, outer central domain; EBa, anterior domain of EB. For genotypes, see Supplemental Information.

Efferent neurons from the bulb region have been shown to target specific ring layers within the EB (R neurons) (Wolff et al., 2015; Franconville et al., 2018). To characterize the matching between TuBu cells and the spatial positioning of R neuron subtype dendrites, we performed a series of co-labeling studies (Figures 7A–7F), which, for technical reasons, focused on two TuBu classes: TuBu-l_p_ and TuBu-c_a_ in combination with different candidate R neuron types of the BU_s_: R2, R4d, and R5. As previously shown, the BU_i_ is innervated by R3 neurons (Figure 7G) but not targeted by TuBu-l or TuBu-c neurons (data not shown, compare (Omoto et al., 2017). In the BU_s_ we could identify matching projection patterns, in which all TuBu axons of one class appeared to contact only one specific R neuron type. This was particularly clear in the case of TuBu-l cells, which clearly overlap with R4d (Figures 7A–7A″) but not with R2 or R5 (Figures 7B–7C‴). For TuBu-c neurons, a partial overlap with the dendritic fields of R2 was detected (Figures 7E and 7E′) while avoiding contacts with R4d and R5 (Figures 7D, 7D′, 7F, and 7F′). Furthermore, co-labeling revealed that dendrites of different R neuron types segregate into coherent, nonoverlapping domains within the bulb neuropil (Figures 7G–7I). In summary, in our analysis of two representative TuBu classes and three candidate R neuron classes innervating the superior bulb (BU_s_), we could dissect one fully matching pair of TuBu → R neuron circuit, as well as another pair with a partial overlap. Thus, yet another synaptic level is added to the parallel visual pathways described here, as distinct AOTU efferents remain separated and contact different EB rings (Figure 7J).

## Discussion

Like various other sensory modalities for which spatial information is critical, neural circuits in the visual system of many animals are organized in a topographic fashion to maintain the neighboring relationship of adjacent pixels detected by photoreceptors in the periphery, along the visual pathways into the central brain (Livingstone and Hubel, 1988). The topographic representation of different kinds of sensory information within the central brain of *Drosophila* is currently being investigated using molecular genetic tools in combination with cell-type-specific driver lines (Tsubouchi et al., 2017; Patella and Wilson, 2018). Although it is well known that spatially patterned visual stimuli induce coherent activity bumps in the Drosophila CX (Seelig and Jayaraman, 2013, 2015; Kim et al., 2017; Green et al., 2017), the pathway translating peripheral visual information into central activity patterns remains poorly understood.

### Parallel Topographic Pathways into the Central Brain

**Figure 8:**
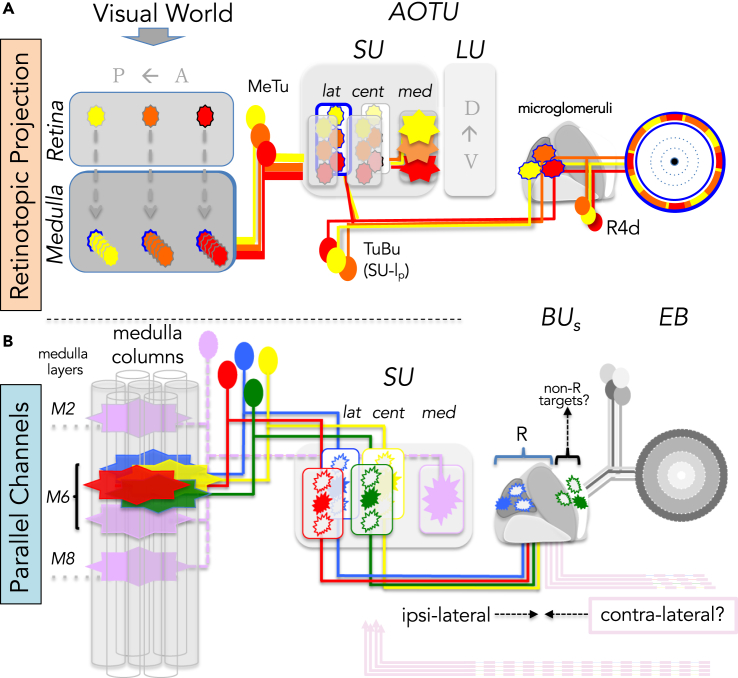
The Anterior Visual Pathway Circuit In the graphic, two features of the pathway—retinotopy and parallel channels—are highlighted. (A) The retinotopy of the pathway is demonstrated by single neurons. Three spatially separate visual stimuli are transmitted by yellow, orange, and red cells, respectively. Innervation patterns in the SU_m_ domain and in the EB indicate a loss of retinotopic arrangements. (B) Parallel channels exist among several synaptic steps. In the medulla, five neuron classes, innervating separate AOTU compartments, detect visual stimuli from the same medulla columns. For two classes, the target areas in the BUs are shown, where corresponding ring neurons (R) transfer the information into the EB. Inhibitory neurons from the opposite hemisphere are possible regulators in the BU_s_ and the AOTU.

Here we have shown that medulla inputs to the AOTU fall into two morphological types regarding their arborization patterns: broad innervation versus spatially restricted axon terminals. In both cases, only a single domain within the AOTU is targeted. Although the topographic representation from the lobula neuropil is mostly lost in the broad innervation pattern of converging and intermingling LC projection neurons onto the majority of optic glomeruli (Panser et al., 2016; Wu et al., 2016; Keles and Frye, 2017), we could identify a unique spatial organization for the output channel from the medulla (Figure 8). Topographic representation of the medulla (at least its dorsal half, where most driver lines used here are expressed) is maintained in the SU of the AOTU, which is spatially separated from lobula representation within the AOTU (the LU). Interestingly, a strict topographic correlation only exists between the a-p position of the dendritic fields of MeTu projection neurons in the medulla and their restricted axon termination along the d-v axis within distinct domains of the SU in the AOTU. No such topography exists along the d-v axis in the medulla. These neurons are therefore well suited for filtering out specific visual information (such as landmarks or celestial bodies) for guiding heading decisions during visually guided navigation (Giraldo et al., 2018).

Based on their morphology, as well as their molecular identity, three principle types of MeTu neurons provide input into the AOTU, with overlapping dendritic fields within the medulla but segregated axon terminals to distinct AOTU (sub-)domains. MeTu-l and -c classes have a similar neuronal morphology with dendrite arborization restricted to a single medulla layer (M6) and spatially narrow axon termination areas in four separate AOTU subdomains (SU-l_a_, -l_p_, -c_a_, and -c_p_), thereby building several pathways arranged in parallel (Figure 8). Our nomenclature of the SU subdomain organization differs slightly from previous studies (Omoto et al., 2017), because it is now based on the expression patterns of different cell surface molecules, which might reflect the functional organization of these structures. Because of this new classification, both lateral and central domains (but not the medial domain) of the SU become further subdivided into anterior (SU-l_a_ and SU-c_p_) and posterior halves (SU-l_p_ and SU-c_p_). Nevertheless, it should be noted that the total number of subdomains remains the same in both nomenclatures, with the major difference being the posterior-lateral subdomain (‘lp’), which has been attributed to the central domain (SU-c_p_) in our study, as part of the Connectin-positive central neuropil. Based on the connectome reconstruction of the hemibrain dataset (Scheffer et al., 2020), which reports in a total number of 347 MeTu neurons (‘MC61-type’), we estimate ∼60 MeTu neurons per topographic class (and twice that for MeTu-m cells), assuming an equal innervation of SU subdomains of similar volume. Because we counted 8–12 TuBu neurons from three independent expression lines, we estimate a convergence ratio from MeTu to TuBu neurons of about 8:1 to 5:1. Only the organization of the MeTu-l and MeTu-c neurons clearly enables a spatial projection of visual information from the columnar organization in the medulla to the corresponding AOTU domains, which seems well suited to relay topographic information along one spatial axis toward the central brain.

### The Transformation of Topographic Information in the Central Brain

The borders of the SU compartments are respected by molecularly defined populations of TuBu neurons, thereby defining the next synaptic elements in the parallel pathways toward the bulb neuropil. Although this neuropil with its afferent (TuBu) and efferent (R neurons) channels has been intensively studied in recent years (Seelig and Jayaraman, 2013, 2015; Omoto et al., 2017; Sun et al., 2017; Shiozaki and Kazama, 2017; Franconville et al., 2018; Green et al., 2017), there still remains a gap in knowledge concerning how precise synaptic connections convey topographic information to the central complex. Four major findings of the TuBu→EB circuit are revealed by our study. First, the topographic position of TuBu dendrites in the SU is not translated into a defined position within the bulb but instead exhibits a targeting plasticity within a restricted bulb area. Secondly, although the recent dissection of the AOTU→EB pathways described the bulb as a tripartite structure (Omoto et al., 2017) including both afferent and efferent neurons, we can now refine this picture by highlighting that although our analysis of TuBu-neurons is mainly restricted to only two representative TuBu classes (one in the SU-l_p_ and the other in the SU-c_a_ domain), both these classes target to areas within the superior bulb (BU_s_). More broadly expressed driver lines revealed exclusive TuBu neuron innervation of the BU_s_, indicating that additional TuBu classes target to this bulb area (data not shown). Thus, we expect at least four different classes of TuBu neurons to exclusively innervate the BU_s_ (TuBu-l_a_, TuBu-l_p_, TuBu-c_a_, and TuBu-c_p_), each of them connecting to a different set of output neurons, indicating an even more complex organization of the bulb, in particular the BU_s_. Thirdly, TuBu classes project onto dendritic areas of R neuron classes (so called “sectors”) within the bulb, and specific connections are formed between TuBu neurons and R neuron classes. Although we could identify three R neuron classes within the BU_s_, there probably exists a much higher diversity of connections within this small area of the bulb, reaching beyond the scope of this study. For instance, the postsynaptic partners of one subset of TuBu-c_a_ neurons as well as neurons contacted by R2 and R5 dendrites remain to be identified. Additional postsynaptic partners other than R neurons are contacted by TuBu neurons, like contralaterally projecting neurons described in the locust (El Jundi and Homberg, 2012) and the bumblebee (Pfeiffer and Kinoshita, 2012), which connect the AOTU units of both hemispheres (TuTu neurons).

It appears therefore that topography is conserved within the AOTU output neuron projections toward the bulb and ring neurons, which is in good agreement with their physiological responses to visual stimuli, like bright objects (Omoto et al., 2017; Sun et al., 2017; Shiozaki and Kazama, 2017). All ring neurons of the same type occupy the same ring layer within the ellipsoid body, raising the question of how topographic information is integrated within central complex neuropils. Interestingly, different MeTu neuron types with similar receptive fields may innervate different AOTU domains and thereby connect to different TuBu neuron populations forming parallel channels that then diverge within the bulb regions, where we found SU-l_p_ and SU-c_a_ efferents mapping onto separate ring neurons (R4d versus R2). Hence, we could define at least two distinct topographic MeTu channels into the central brain. Although functional differences between the BU_i_ and BU_s_ have been described (Omoto et al., 2017), functional studies (Seelig and Jayaraman, 2013; Sun et al., 2017) have not yet compared the physiological responses of different TuBu classes or the responses of R neurons within the BU_s_. Based on the data presented here, we would expect that retinotopic information in the BU_s_ remains represented in the respective sector that is associated with their TuBu class.

### An Additional, Nontopographic Pathway into the Central Brain

A morphologically distinct class of MeTu cells is formed by MeTu-m cells. One distinguishing feature in respect to other MeTu cell types is that many cells arborize broadly in their respective AOTU domain. We found axon terminals of single MeTu-m neurons invariably spread across the a-p axis of their SU-domain, whereas in the d-v axis they either covered their domain completely or partially—the former case being reminiscent of the afferent organization of LC neurons from the lobula within optic glomeruli in the PVLP regions, whereas the latter case is similarly described for lobula neurons innervating the AOTU's large unit (LU) (Wu et al., 2016), where the topography of LC10 neurons in the LU has been analyzed, resulting in the distinction of four different LC10-classes. It remains to be seen whether MeTu-m neurons also could be divided into such classes. Those cells innervating the complete SU-m are well suited to form a nontopographic channel to the central brain. Interestingly, although topographic MeTu-l and -c neurons form dendritic fields within a single medulla layer, MeTu-m neurons integrate from three different medulla layers, reminiscent and in fact similar to some lobular LC neuron types, the main afferents of the AOTU large unit, for which a comparable rough topography along the dorsoventral axis has previously been found (Wu et al., 2016). Furthermore, only MeTu-m neurons form a collateral arborization in the lobula, indicating that this pathway could directly integrate visual information from both the medulla and lobula. Our observation that MeTu-m neurons contact a population of TuBu neurons that projects into the inferior bulb area (Bu_i_) separated from other TuBu neurons further suggests a different role for this pathway. Sun et al. (2017) describe a contralateral inhibition mediated by the Bu_i_, supporting a model in which the SU-m pathway is involved in suppressing ipsilateral stimuli with the expense of reduced spatial resolution.

Taken together, topographic and nontopographic afferents generate an interesting assembly of adjacent domains within the AOTU, from exclusively topographic medulla input in SU-l and SU-c domains, nontopographic medullar (and potentially also lobular) input in SU-m, and another large area of nontopographic input exclusively from the lobula in the LU (Figure 8). Thus, we have identified multiple parallel topographic pathways separated from a parallel nontopographic channel.

### Evolutionary Conservation of the Anterior Visual Pathway

This principle visual pathway involving the AOTU as a central relay station between medullar/lobular inputs and the central brain is widely shared among different insect taxa, where homologous structures can be found, e.g. orthopterans (Homberg et al., 2003), hymenopterans (Mota et al., 2011), and beetles (Immonen et al., 2017). The stimuli conveyed by this “anterior visual pathway” have been addressed in only a few insect species so far. Most prominently, the AOTU has been associated with celestial orientation using polarized skylight in several species (Pfeiffer et al., 2005) or in chromatic processing (Paulk et al., 2008; Mota et al., 2013). Dorsal rim ommatidia harboring polarization-sensitive photoreceptors for polarized light vision are crucial for the sky-compass orientation and exist in most insects analyzed, such as locusts (Pfeiffer et al., 2005; Homberg and Paech, 2002), butterflies (Heinze and Reppert, 2011; Labhart et al., 2009), and honeybees (Held et al., 2016), as well as flies (Wada, 1971, 1974; Wernet et al., 2003). However, it remains unknown whether MeTu neurons receive direct or indirect input from modality-specific cell types located in the DRA (Sancer et al., 2019, 2020). In addition, processing of chromatic information was also shown to be accomplished via the AOTU in several insects (Otsuna et al., 2014; Mota et al., 2013). We have now identified inputs to this pathway, by identifying direct connections between MeTu cells and UV-sensitive R7 photoreceptor cells in medulla layer M6.

Furthermore, the molecular markers used here can serve as future tools to reveal the molecular mechanisms that underlie the formation of the LC-optic glomeruli network across species. Because *Drosophila* is among the smallest species for which the AOTU has been characterized and is believed to be a behavioral generalist, even more sophisticated architectures of the SU-homologue could exist in other insect taxa. On the anatomical and functional level, optic glomeruli share many features with the synaptic neuropil within the antennal lobe, which led to the postulation that the glomerular organization in the protocerebrum (optic glomeruli) and the deutocerebrum (olfactory glomeruli) are in fact homologous structures (Strausfeld, 1989; Mu et al., 2012). Indeed, we found molecular characteristics in the PVLP and AOTU that resemble the combinatorial code of cell-surface proteins in the olfactory system (e.g. expression patterns of Ten-m, Con, Caps, and Sema1a in both systems). However, future developmental studies of mutant LC and MeTu neurons are needed to test to what extent common mechanisms of glomerular circuit assembly exist in both sensory systems. Although the idea of a serial homology of glomerular organized neural system is far from being resolved, it will be intriguing for further studies to analyze the developmental mechanisms that underlie the circuit formation of these parallel AOTU pathways and optic glomeruli circuits as well as to compare them with known molecular functions during olfactory system maturation.

### Limitations of the Study

We cannot exclude the fact that the SU of the AOTU might consist of additional functional units that so far have not been identified and that we missed neurons in our analysis due to the lack of expression lines to visualize them. Populations of neurons that we classified as a single type might turn out to be different enough (by morphology and/or synapse partners) to justify the establishment of further pathways, and we might have missed these cell types in our single cell labeling experiment, as this method involves random events where scarcer neurons can easily remain unnoticed. *In vivo* experiments measuring neuronal activity and responses to visual stimuli were beyond the scope of our study but will be an essential part for understanding the functional features of the circuit. The wealth of genetic tools and their manifold combinations in Drosophila certainly provide capabilities of detailed analyses. As the driver lines we used for our study to unravel the components of the visual pathway are publicly available and could be used to measure and manipulate neuronal activity, we hope to have paved the way for future studies of components of this visual circuit.

### Resource Availability

#### Lead Contact

Further information and requests for resources and reagents should be directed to and will be fulfilled by the Lead Contact, Thomas Hummel (thomashummel@univie.ac.at).

#### Materials Availability

The ort-mCD8::GFP construct is available on request without restriction.

#### Data and Code Availability

This study did not generate datasets or analyze codes.

## Methods

All methods can be found in the accompanying Transparent Methods supplemental file.

## Supplemental Information

Document S1. Transparent Methods and Figures S1–S5

## Footnotes

Supplemental Information can be found online at https://doi.org/10.1016/j.isci.2020.101590.
