# Supplementary material for: Parallel Visual Pathways with Topographic versus Nontopographic Organization Connect the Drosophila Eyes to the Central Brain
Source: iScience. 2020 Sep 19;23(10):101590. doi: 10.1016/j.isci.2020.101590 (PMC7648135; doi:10.1016/j.isci.2020.101590)
Supplement: Document S1. Transparent Methods and Figures S1–S5 [file mmc1.pdf]

iScience, Volume *23*

Supplemental Information

Parallel Visual Pathways with Topographic versus Nontopographic Organization Connect the *Drosophila* Eyes to the Central Brain

Lorin Timaeus, Laura Geid, Gizem Sancer, Mathias F. Wernet, and Thomas Hummel

Supplemental Figures

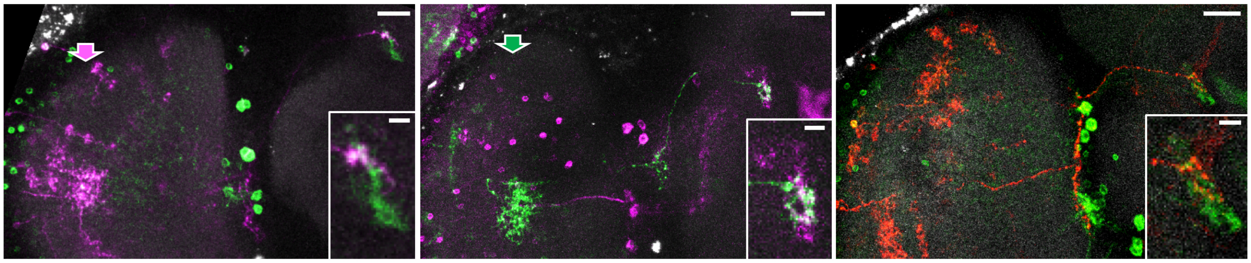

Figure S1. Morphology of MeTu-m neurons. Related to Fig. 1. FLYBOW clones of MeTu-m neurons with restricted innervation of the SU-m domain. Approximate center of dendritic area in the medulla is indicated by colored arrows. In the last image, the area of medullar innervation could not be resolved. The innervated area of the SU domain is magnified in the insets. Scale bars, 20μm and 5μm (Insets). Genotype: *hs-mFlp5; R20B05>FLYBOW1.1*.

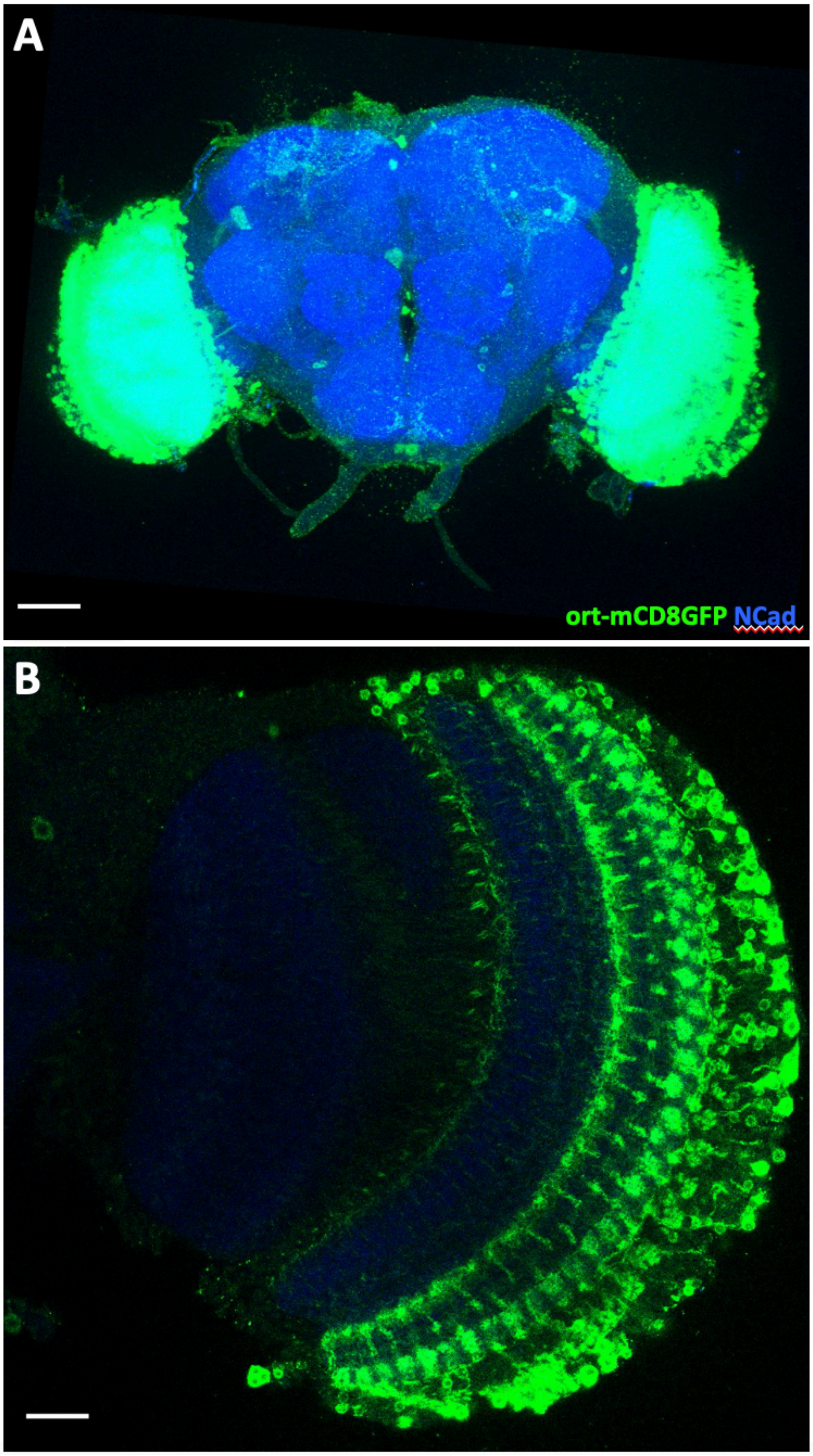

Figure S2. Expression pattern of the ort-mCD8GFP construct. Related to Fig. 3. A. Overview of neurons in the brain labeled by the ort-insertion. Scale bar, 50µm. **B.** Expression of ort-mCD8GFP in the medulla. Scale bars, 50µm (A); 20µm (B). Genotype: *ort-mCD8GFP*.

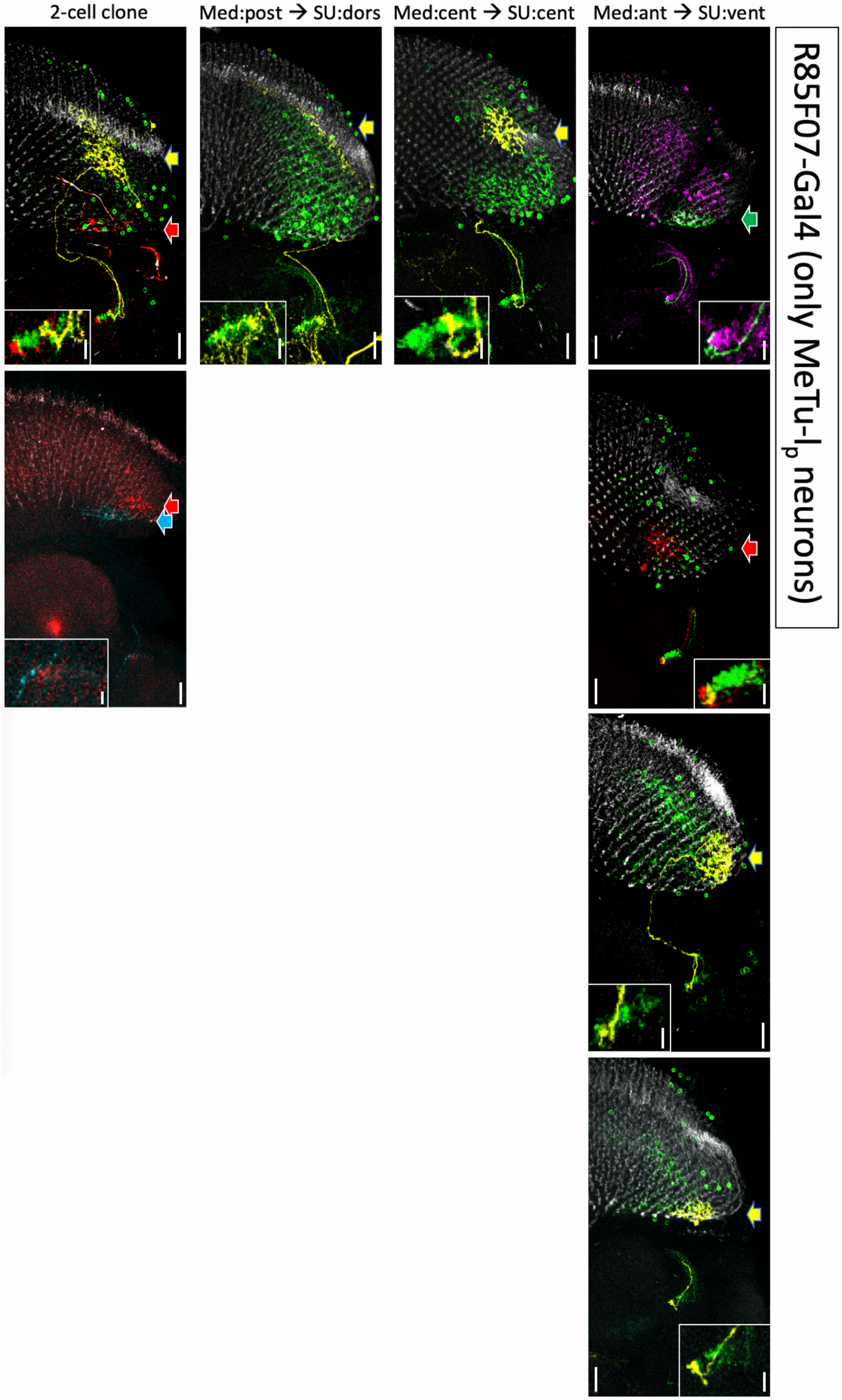
2-cell clone
Med:post → SU:dors
Med:cent → SU:cent
Med:ant → SU:vent
R85F07-Gal4 (only MeTu-I_p neurons)

Figure S3. Topographic relations (medulla → SU) of FLYBOW single or two cell clones in a driver line labeling MeTu-I_p neurons. Related to Fig. 4. Three categories of dendritic (anterior, central, posterior) and axon terminal position (dorsal, central, ventral) where chosen for the medulla and the SU, respectively. The approximate center of the dendritic area (in a-p axis) is indicated by the colored arrows. Cell pairs in the same color where included in the study when their dendritic areas where in close proximity to each other. R85F07-Gal4 exclusively labels MeTu neurons innervating the SU-I_p. The innervated area of the SU domain is magnified in the insets. Scale bars, 20μm and 5μm (Insets). Genotype: *hs-mFlp5; R85F07>FLYBOW1.1*.

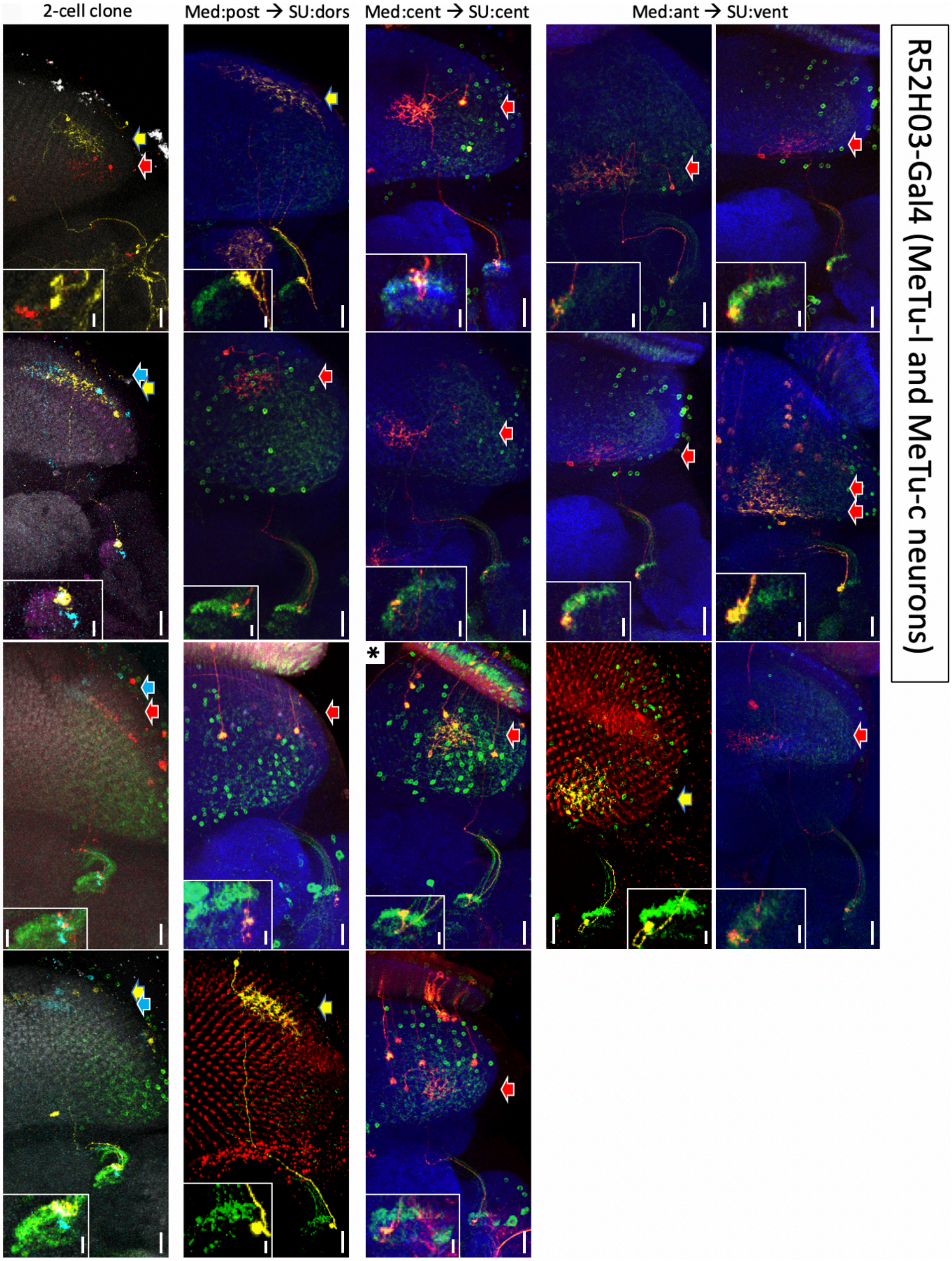
2-cell clone
Med:post → SU:dors
Med:cent → SU:cent
Med:ant → SU:vent
*
R52H03-Gal4 (MeTu-l and MeTu-c neurons)

Figure S4. Topographic relations (medulla → SU) of FLYBOW single or two cell clones in a driver liner labeling MeTu-l and -c neurons. Related to Fig. 4. Compare legend to Fig. S3 for more information. The driver line R52H03-Gal4 labels most MeTu-l and MeTu-c neurons. Different cell populations where not distinguished in this analysis. An asterisk marks the sample with the highest number of medullar columns covered in the central medulla (see main text). Genotype: *hs-mFlp5; R52H03>FLYBOW1.1*.

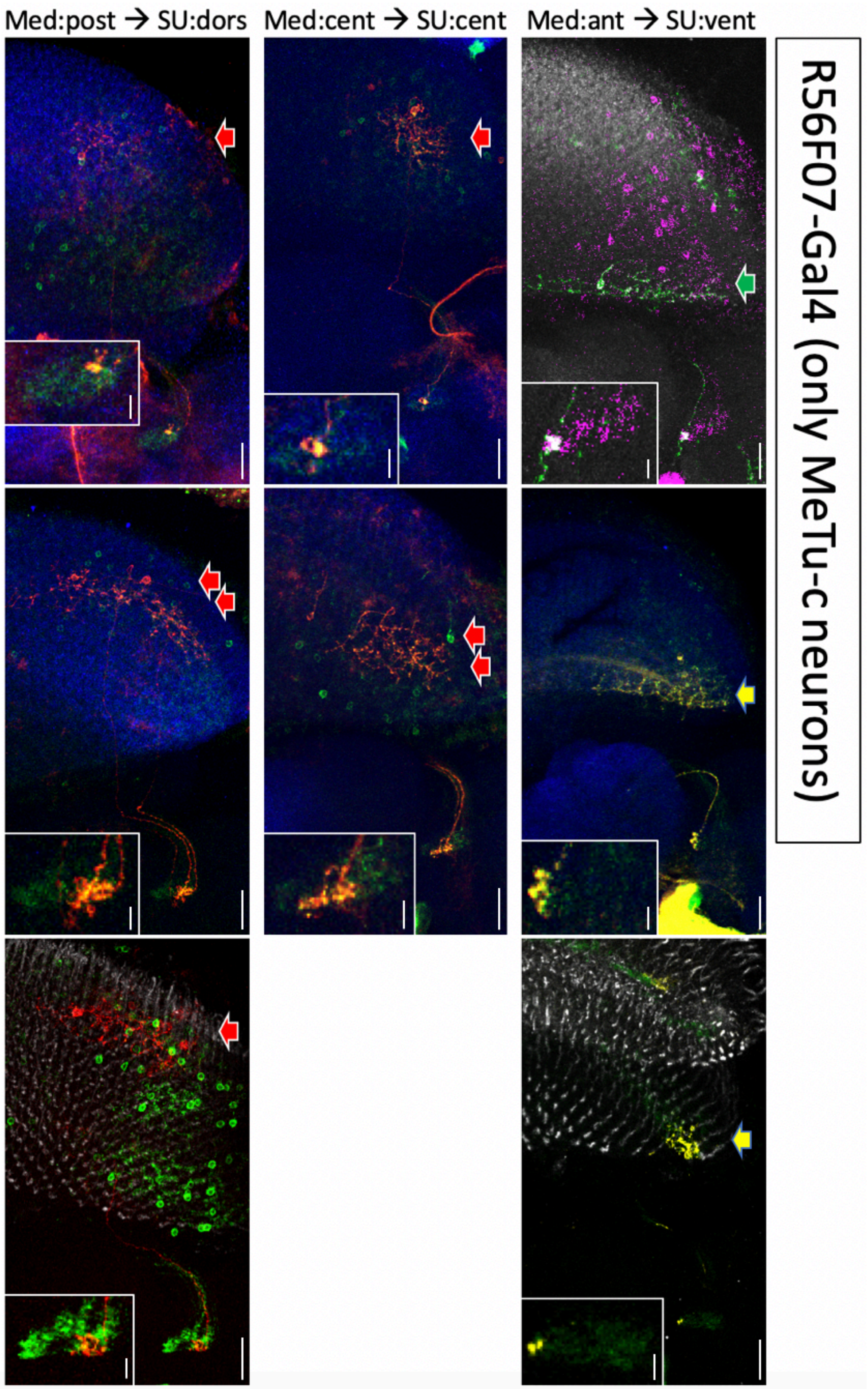

Figure S5. Topographic relations (medulla → SU) of FLYBOW single cell clones in a driver liner labeling MeTu-c neurons. Related to Fig. 4. Compare legend to Fig. S3 for more information. Populations of MeTu neurons labeled by R56F07-Gal4 innervate the SU-c_a and SU-c_p domains. Genotype: *hs-mFlp5; R56F07>FLYBOW1.1*.

Transparent Methods

Fly rearing

Flies were maintained in vials containing standard fly food medium at 25°C at 60% relative humidity unless otherwise mentioned. Canton-S flies were used as a wild type strain.

Fly stocks

Visual circuit analysis was largely based on commercially available enhancer-fragment driver lines. The following lines were generated at the Fly Light Gal4-/LexA-Collection (Jenett et al., 2012) and obtained from Bloomington Drosophila Stock Center (BDSC). One driver line was obtained from the Vienna Tiles (VT) collection (Kvon et al., 2014).

	Gal4	LexA	labeling purpose
OPTIC GLOMERULI:			
	R41C07		LC06
	R35D04		LC12 & LC10
		VT29314	LC10
METU-NEURONS:			
	R52H03		MeTu-l & MeTu-c
	R85F05		MeTu-l
		R94G05	MeTu-l
	R44A03		MeTu-c
		R67C09	MeTu-c
	R25H10		MeTu-c
	R56F07		MeTu-c
	R20B05	R20B05	MeTu-m
TUBU-NEURONS:			
	R86C02		TuBu
	R71E07		TuBu-l & TuBu-c
	R25F06	R25F06	TuBu-l
	R64F06	R64F06	TuBu-c
R-NEURONS:			
	R14A12	R14A12	R3
	R12B01		R4d
		R85E07	R4d
	R49B02		R5
		R48H04	R5

Stocks for clonal analysis and effector lines for cell labeling:

FRT42D; FRT42D TubP-Gal80; UAS-mCD8::GFP and UAS-mCherry strains were obtained from BDSC. The UAS-DenMark construct was provided by Bassem Hassan, LexAop::GFP was a gift from Andrew Straw. Flies for synaptic-GRASP experiments (UAS-Syb::spGFP1-10

& LexAop spGFP11::CD4) (Karuppudurai et al., 2014) were a gift from Chi-Hon Lee. The FLYBOW components where provided by Iris Salecker.

Generation of ort-mCD8 transgenic flies

A ~3.5 fragment from the ort gene spanning the entire 5' intergenic region, as well as the 1st untranslated exon and the transcription start was PCR-amplified, with appropriate restriction endonuclease recognition sites attached to the primers. The fragment was subcloned, sequenced and ligated into a promoterless injection vector (pCasper-mCD8:GFP-SV40). Insertions on 2nd and 3rd chromosomes were obtained via commercial embryo injection. Interestingly, expression was not variegated as seen for many ort-Gal4 constructs. Further information is available upon request.

Specific cell labeling:

In addition to the enhancer-fragment expression lines listed above, these lines were used to visualize specific neuron types: Or67d::GFP and OR67d-Gal4 (Couto et al., 2005) were used for olfactory class visualization, glia cells were marked by repo-Gal4, and Chi-Hon Lee provided the ortC1a-LexA::VP16 (Ting et al., 2014) construct for labeling of Dm8 neurons. PanR7-Gal4 (rh3+rh4-Gal4) was used for R7 TransTango, and panR8-Gal4 (rh5+rh6-Gal4) for R8 TransTango experiments (both gifts from Claude Desplan). Caps-Gal4 (Shinza-Kameda et al., 2006) was used in a MARCM background to visualize different optic glomeruli.

<u>Antibodies used in this study</u>:

Primary antibodies used were: 24B10/Mouse anti-Chaoptin (1:50, DSHB), DN-Ex #8/Rat anti-CadN (1:20, DSHB), Flamingo#74/Mouse anti-flamingo (1:20, DSHB), Rabbit anti-GFP (1:1000, Invitrogen, Carlsbad, CA). Mouse anti-Teneurin-m (1:20) was a kind gift from Stefan Baumgartner, anti-Connectin (1:20) was kindly provided by Robert AH White.

Secondary antibodies used: Goat anti-Rabbit Alexa-488 (1:500), Goat anti-Rabbit Alexa-568 (1:300), Goat anti-Mouse Alexa-488 (1:300), Goat anti-Mouse Alexa-647 (1:500), Goat anti-Rat Alexa-647 (1:300). All secondary antibodies were obtained from ThermoFisher Scientific (Alexa Fluor®, Molecular Probes™).

Clonal analysis

Two approaches for visualization of large and small genetic mosaics were used respectively. For inducing larger mosaics, MARCM clones with an ey-Flp insertion on the X chromosome were generated (Lee and Luo, 1999). This approach was possible because Flp under the control of the ey-promoter is not only expressed in peripheral sensory neurons, but we found it also to be active in medulla and lobula projecting neurons innervating the optic glomeruli. For small clones and single-cell analysis, we used the temperature-sensitive hs-mFlp5 promotor in combination with a FLYBOW (FB1.1B)-construct (Hadjieconomou et al., 2011, Shimosako, Hadjieconomou et al., 2014). Prior to screening for brains with single cell labeling, a heat shock was given to developing flies (L2-stage, L3-stage, early pupal) for 30min, 1h or 2h at 38°C. The exact timing protocol was under undergoing adjustment for each experiment. The pupae were then allowed to further develop at 25°C and dissected within two days after eclosion.

Immunohistochemistry

Drosophila brains were dissected in phosphate-buffered saline (PBS) and fixed in 4% paraformaldehyde (PFA) in PBS for 20 min. Samples were washed 3 x 15 min with PBST (PBS containing 0.3 % Triton X-100) and blocked for 3 hours (10% Goat serum in PBST) under constant shaking on a horizontal shaker, before incubating in primary antibody solution for two days at 4°C. Washing procedure was repeated before incubating with secondary antibody for two days at 4°C. Following three times washing, the brains were mounted in Vectashield® (Vector Laboratories, Burlingame, CA) anti-fade mounting medium prior to confocal microscopy. Images were obtained using a TCS SP5II confocal microscope (Leica) using 20x and 63x glycerol immersion objectives. Image processing was performed using ImageJ and Adobe Photoshop® CS6.

Activity GRASP

Flies were grown in a 12h-12h dark-light cycle incubator at 25°C in normal vials. 1-day old flies were kept in a 25°C, 20 h – 4 h light-dark cycle custom-made light box for 3 days to ensure sufficient activation of visual neurons. Brains were stained with polyclonal GFP (anti GFP goat pAB) and monoclonal GFP (anti-GFP rat mAB) antibody to visualize pre-synaptic cells and GRASP signal, respectively. Post-synaptic cells were visualized by staining with CD4 antibody.

TransTango

Flies for TransTango experiments were either kept in 18°C, in a 12h-12h dark-light cycle incubator and dissected when they were 15 days old.

Drosophila genotypes used in the respective figures

Figure 1

B) *OR47d::GFP*, C) *hs-mFlp5; OR67d-Gal4>FLYBOW1.1*, E) *ey-Flp; FRT40, Gal80/FRT40*; *Caps-Gal4>mCD8::GFP* (MARCM), F) *R41C07-Gal4>mCherry, >syt::GFP*, G) *hs-mFlp5*; *R41C07-Gal4>FLYBOW1.1*, H) *R35D04-Gal4>DenMark, >syt::GFP*, J) *hs-mFlp5; R35D04-Gal4>FLYBOW1.1,* K, L) *VT29314-LexA>mCD8::GFP; R44A03-Gal4>mCherry*, N) *hs-mFlp5; R52H03-Gal4>FLYBOW1.1*, O) *hs-mFlp5; R20B05-Gal4>FLYBOW1.1*

Figure 2

B) *Repo-Gal4>mCD8::GFP*, E) *Caps-Gal4>mCD8::GFP*, F, F') *R85F05-Gal4>mCD8::GFP*, G, G') *R20B05-LexA>mCD8::GFP*, H) *R25H10-Gal4>mCherry; R67C09-LexA>mCD8::GFP*, J) *R85F05-Gal4>mCD8::GFP*, K) *R44A03-Gal4>mCD8::GFP*, L) *R56F07-Gal4>mCherry, Dm8-LexA>mCD8::GFP*, M) *R20B05-LexA>mCD8::GFP,* N, N') *R56F07-Gal4>mCherry, R20B05-LexA>mCD8::GFP*, O, O') *R56F07-Gal4>mCherry, R20B05-LexA>mCD8::GFP*, P, P') *R44A03-Gal4>mCherry, R94G05-LexA>mCD8::GFP*

Figure 3

A) *panR7-Gal4>transTango*, B) *panR8-Gal4>transTango*, C) *R94G05-Gal4>MCFO-1*, D) *ort-mCD8::GFP*, E) *ort-mCD8::GFP; R94G05-Gal4>myrTomato*, F) *ort-mCD8GFP; R52H03-Gal4>myrTomato*, G) *ort-mCD8::GFP; R67C09-Gal4>myrTomato*, H) *ort-mCD8::GFP; R25H10-Gal4>myrTomato*, I) *ort-mCD8::GFP; R20B05-Gal4>myrTomato*

Figure 4

A, B) *hs-mFlp5; R52H03-Gal4>FLYBOW1.1*, C) *hs-mFlp5; R20B05-Gal4>FLYBOW1.1*, D-F) *hs-mFlp5; R52H03-Gal4>FLYBOW1.1*, G-J) *hs-mFlp5; R56F07-Gal4>FLYBOW1.1*, K-M) *hs-mFlp5; R85F05-Gal4>FLYBOW1.1*

Figure 5

A) *R86C02-Gal4>DenMark, >syt::GFP*, B) *R25F06-LexA>GFP; R64F06-Gal4>mCherry*, C) *R25F06-LexA>GFP*, C') *R85F05-Gal4>syb::spGFP1-10; R25F06-LexA>CD4::spGFP11*, D) *R64F06-LexA>GFP*, D') *R56F07> syb::spGFP1-10; R64F06-LexA>CD4::spGFP11*, D'') *R85F05-Gal4>syb::spGFP1-10; R64F06-LexA>CD4::spGFP11,* F-J) *hs-mFlp5; R86C02-Gal4>FLYBOW1.1*

Figure 6

A) *hs-mFlp5; R71E07-Gal4>FLYBOW1.1*, B) *hs-mFlp5; R86C02-Gal4>FLYBOW1.1*, C) *hs-mFlp5; R64F06-Gal4>FLYBOW1.1*, D, F) *R25F06-Gal4>FLYBOW1.1*

Figure 7

A) *R25F06-LexA>GFP; R12B01-Gal4>mCherry*, B) *R25F06-LexA>GFP; EB1-Gal4>mCherry*, C) *R25F06-LexA>GFP; R49B02-Gal4>mCherry*, D) *R64F06-LexA>GFP; R12B01-Gal4>mCherry*, E) *R64F06-LexA>GFP; EB1-Gal4>mCherry*, F) *R64F06-LexA>GFP; R49B02-Gal4>mCherry*, G) *R14A12-LexA>GFP; R12B01-Gal4>mCherry*, H) *R48H04-LexA>GFP; EB1-Gal4>mCherry*, J) *R85E07-LexA>GFP; EB1-Gal4>mCherry*

Supplemental References

Couto, A., Alenius, M. and B. J. Dickson (2005). “Molecular, anatomical, and functional organization of the Drosophila olfactory system.” Curr Biol 15(17): 1535-1547.

Hadjieconomou, D., S. Rotkopf, C. Alexandre, D. M. Bell, B. J. Dickson and I. Salecker (2011). "Flybow: genetic multicolor cell labeling for neural circuit analysis in Drosophila melanogaster." Nat Methods 8(3): 260-266.

Jenett, A., G. M. Rubin, T. T. Ngo, D. Shepherd, C. Murphy, H. Dionne, B. D. Pfeiffer, A. Cavallaro, D. Hall, J. Jeter, N. Iyer, D. Fetter, J. H. Hausenfluck, H. Peng, E. T. Trautman, R. R. Svirskas, E. W. Myers, Z. R. Iwinski, Y. Aso, G. M. DePasquale, A. Enos, P. Hulamm, S. C. Lam, H. H. Li, T. R. Laverty, F. Long, L. Qu, S. D. Murphy, K. Rokicki, T. Safford, K. Shaw, J. H. Simpson, A. Sowell, S. Tae, Y. Yu and C. T. Zugates (2012). "A GAL4-driver line resource for Drosophila neurobiology." Cell Rep 2(4): 991-1001.

Karuppudurai, T., T. Y. Lin, C. Y. Ting, R. Pursley, K. V. Melnattur, F. Diao, B. H. White, L. J. Macpherson, M. Gallio, T. Pohida and C. H. Lee (2014). "A hard-wired glutamatergic circuit pools and relays UV signals to mediate spectral preference in Drosophila." Neuron 81(3): 603-615.

Kvon, E. Z., T. Kazmar, G. Stampfel, J. O. Yanez-Cuna, M. Pagani, K. Schernhuber, B. J. Dickson and A. Stark (2014). "Genome-scale functional characterization of Drosophila developmental enhancers in vivo." Nature 512(7512): 91-95.

Lee, T. and L. Luo (1999). "Mosaic analysis with a repressible cell marker for studies of gene function in neuronal morphogenesis." Neuron 22(3): 451-461.

Shimosako, N., D. Hadjieconomou and I. Salecker (2014). "Flybow to dissect circuit assembly in the Drosophila brain." Methods Mol Biol 1082: 57-69.

Shinza-Kameda, M., E. Takasu, K. Sakurai, S. Hayashi and A. Nose (2006). "Regulation of layer-specific targeting by reciprocal expression of a cell adhesion molecule, capricious." Neuron 49(2): 205-213.

Ting, C. Y., P. G. McQueen, N. Pandya, T. Y. Lin, M. Yang, O. V. Reddy, M. B. O'Connor, M. McAuliffe and C. H. Lee (2014). "Photoreceptor-derived activin promotes dendritic termination and restricts the receptive fields of first-order interneurons in Drosophila." Neuron 81(4): 830-846.
